# Supplementary material for: A Brand-New Metal Complex Catalyst-Free Approach to the Synthesis of 2,8-Dimethylimidazo[1,2-b]pyridazine-6-Carboxylic Acid—A Key Intermediate in Risdiplam Manufacturing Process
Source: Molecules. 2025 Jul 18;30(14):3011. doi: 10.3390/molecules30143011 (PMC12300203; doi:10.3390/molecules30143011)
Supplement: Supplementary file 1 [file molecules-30-03011-s001.zip › molecules-3665157-supplementary.pdf]

ELECTRONIC SUPPLEMENTARY MATERIAL

A Brand-New Metal Complex Catalyst-Free Approach to the Synthesis of 2,8-Dimethylimidazo[1,2-*b*]pyridazine-6-Carboxylic Acid—A Key Intermediate in Risdiplam Manufacturing Process

Georgiy Korenev *, Alexey A. Gutenev, Fyodor V. Antipin, Vladimir V. Chernyshov, Maria P. Korobkina, Maxim B. Nawrozkij and Roman A. Ivanov

Medicinal Biotechnology Department, Sirius University of Science and Technology, Olimpiyskiy Ave. 1, 354340 Sirius, Krasnodar Region, Russia; vladimir.chernyshov2012@yandex.ru (V.V.C.) ivanov.ra@talantiuspeh.ru (R.A.I.)

* Correspondence: korenev.g@talantiuspeh.ru

Table of Contents

Copies of NMR spectra 3
Copies of HRMS spectra 31

Copies of NMR spectra

¹H NMR spectrum of 5-methyl-6-oxo-1,6-dihydropyridazine-3-carboxylic acid **17**

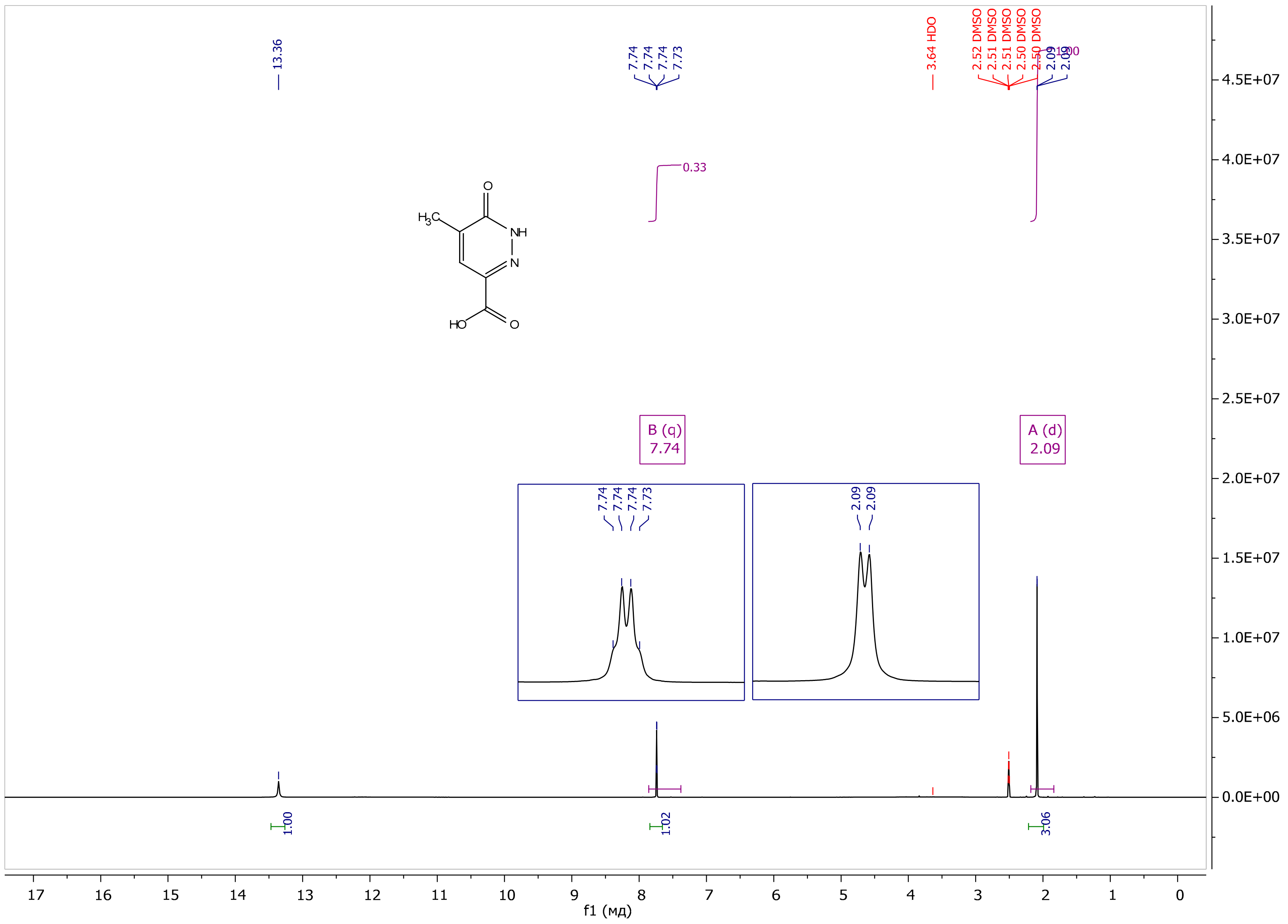

^{13}C NMR spectrum of 5-methyl-6-oxo-1,6-dihydropyridazine-3-carboxylic acid **17**

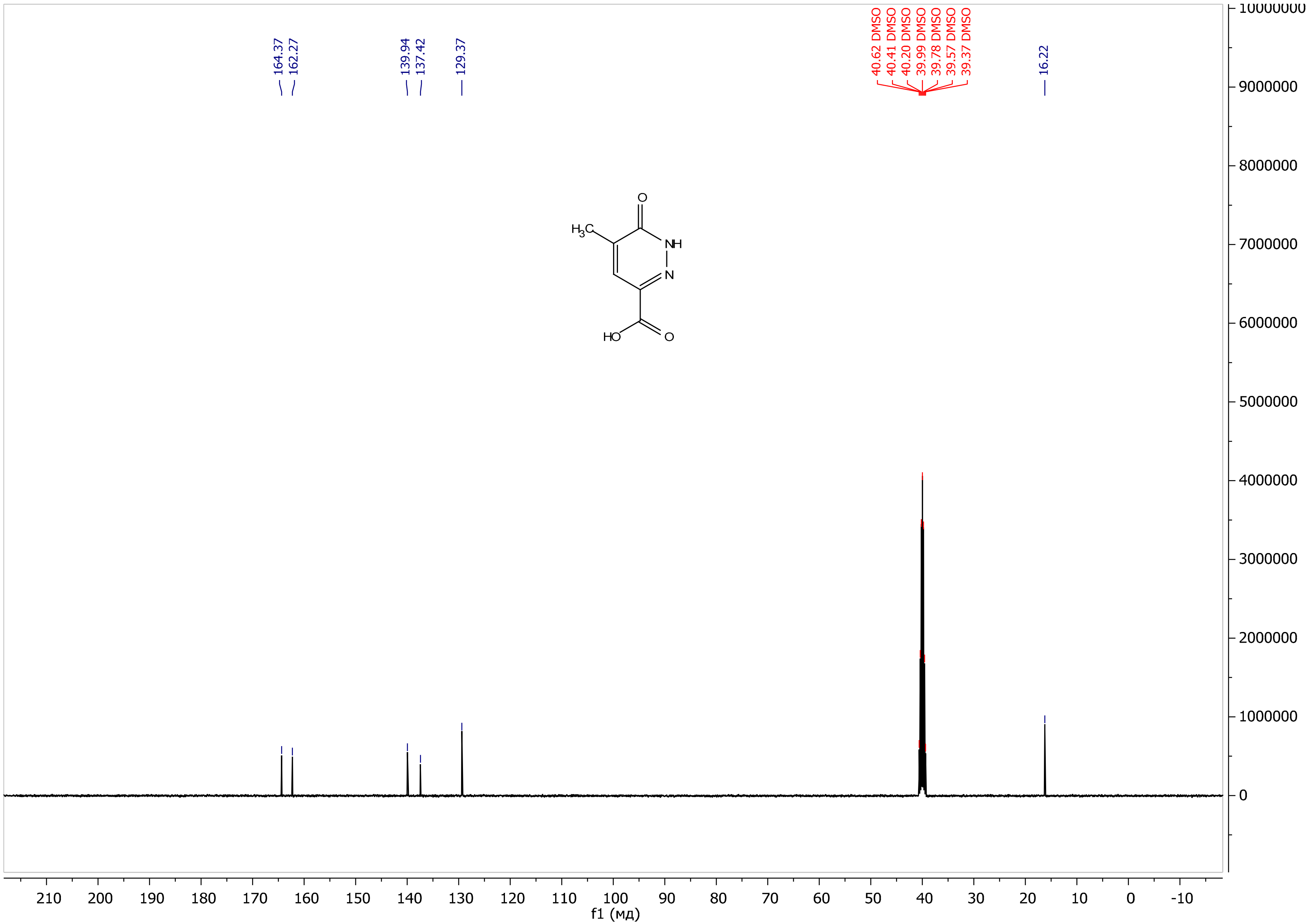

^{1}H NMR spectrum of ethyl 5-methyl-6-oxo-1,6-dihydropyridazine-3-carboxylate **18**

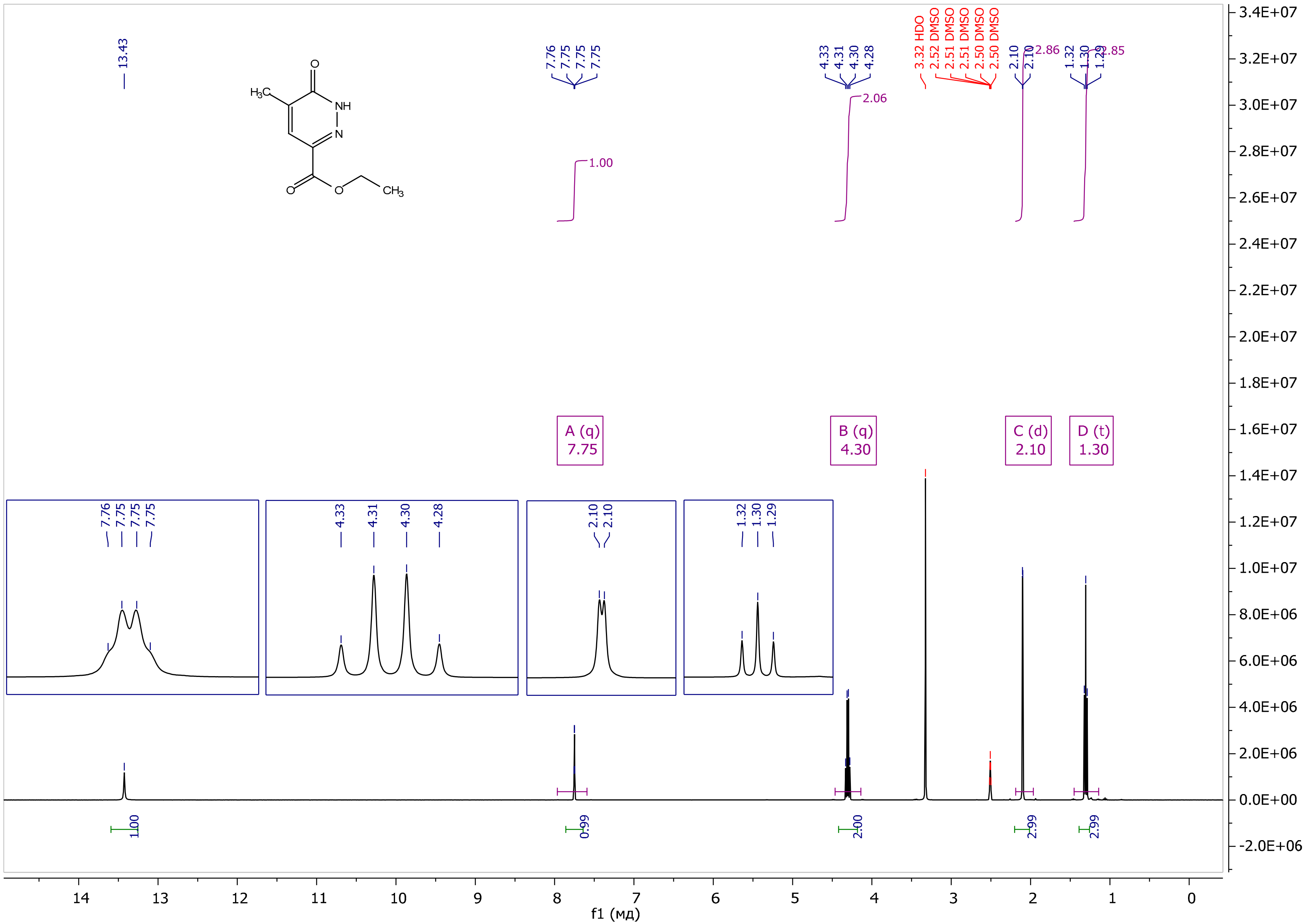

^{13}C NMR spectrum of ethyl 5-methyl-6-oxo-1,6-dihydropyridazine-3-carboxylate **18**

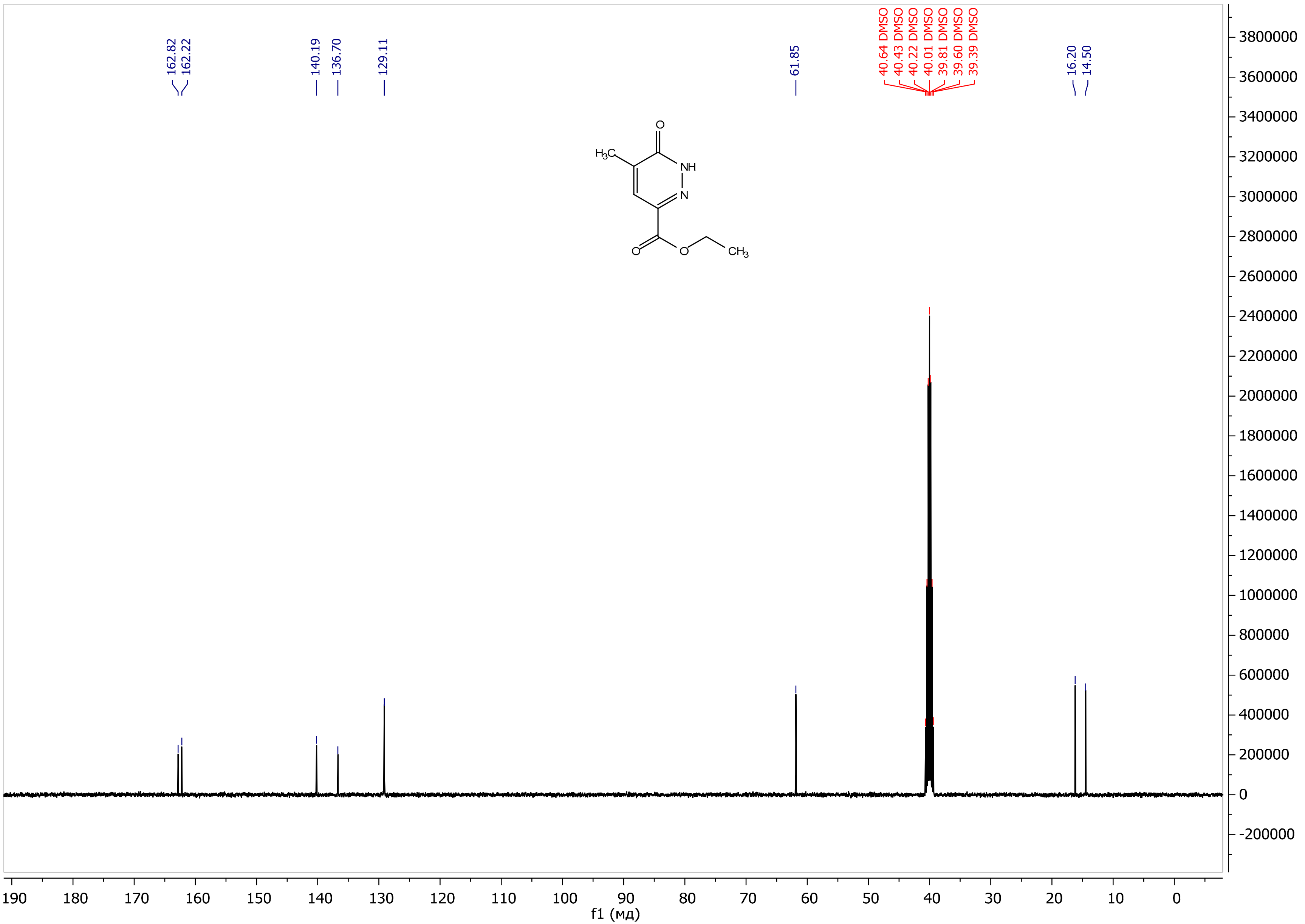

^{1}H NMR spectrum of ethyl 6-chloro-5-methylpyridazine-3-carboxylate **19**

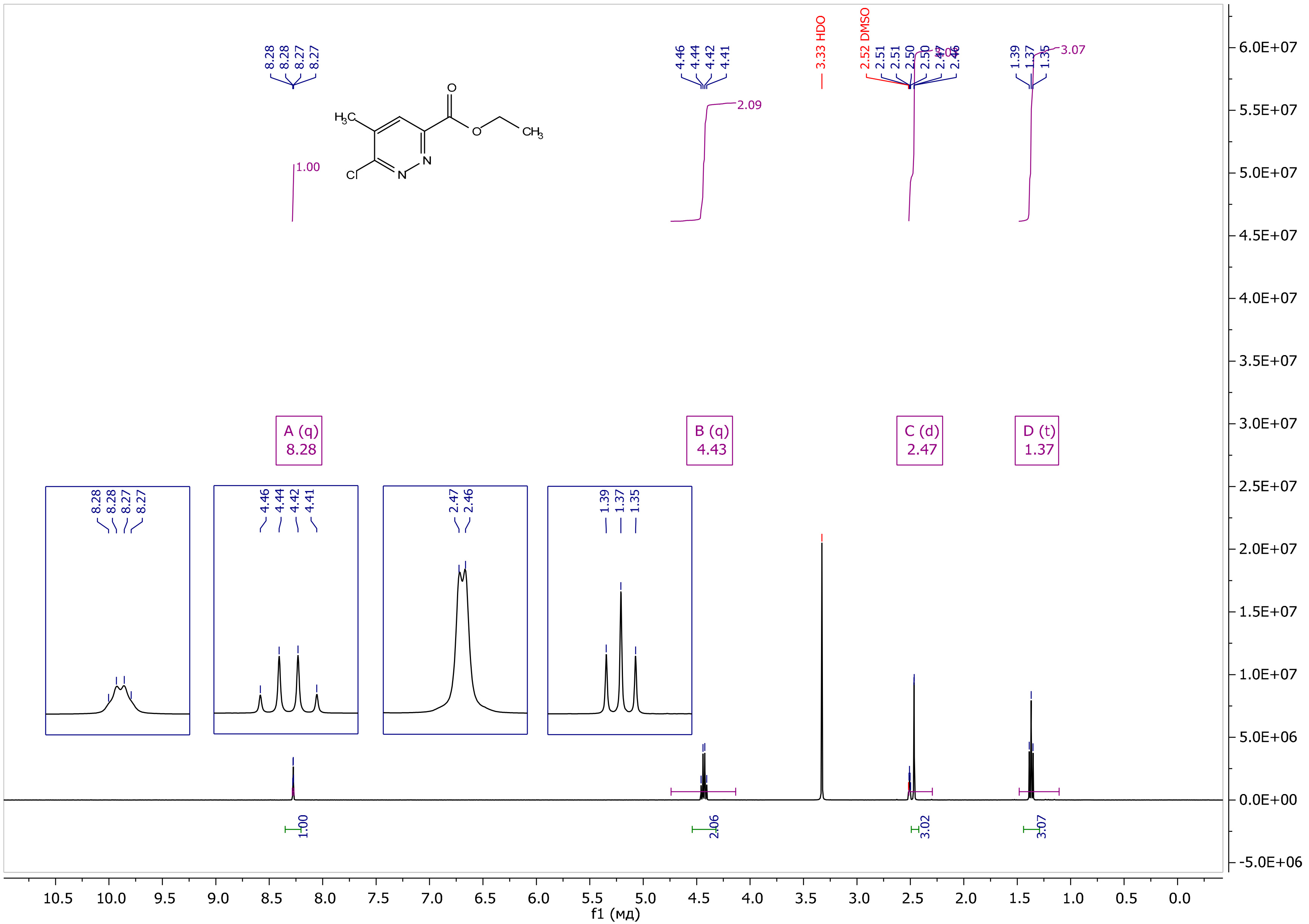

^{13}C NMR spectrum of ethyl 6-chloro-5-methylpyridazine-3-carboxylate **19**

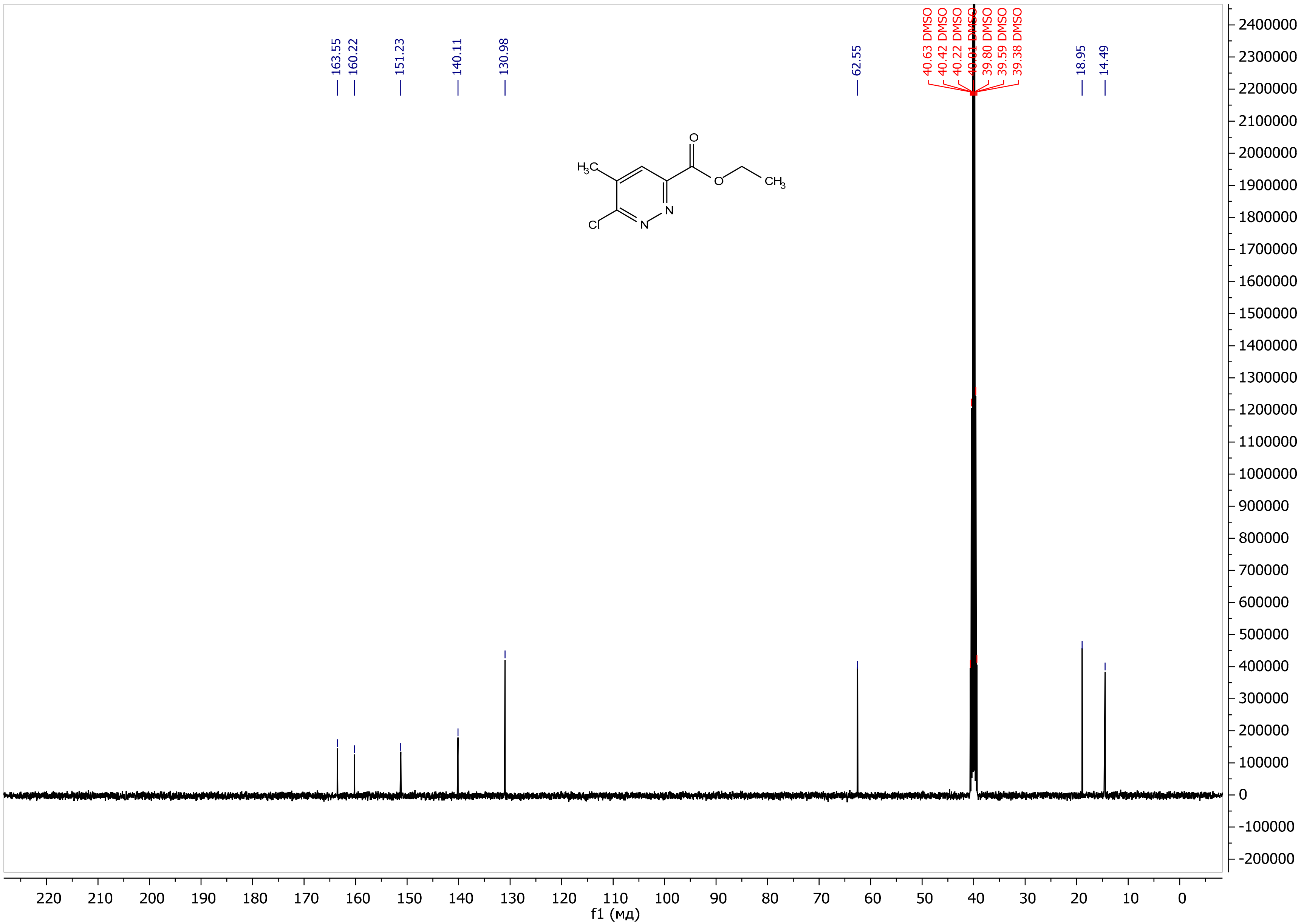

^{1}H NMR spectrum of ethyl 6-azido-5-methylpyridazine-3-carboxylate **20**

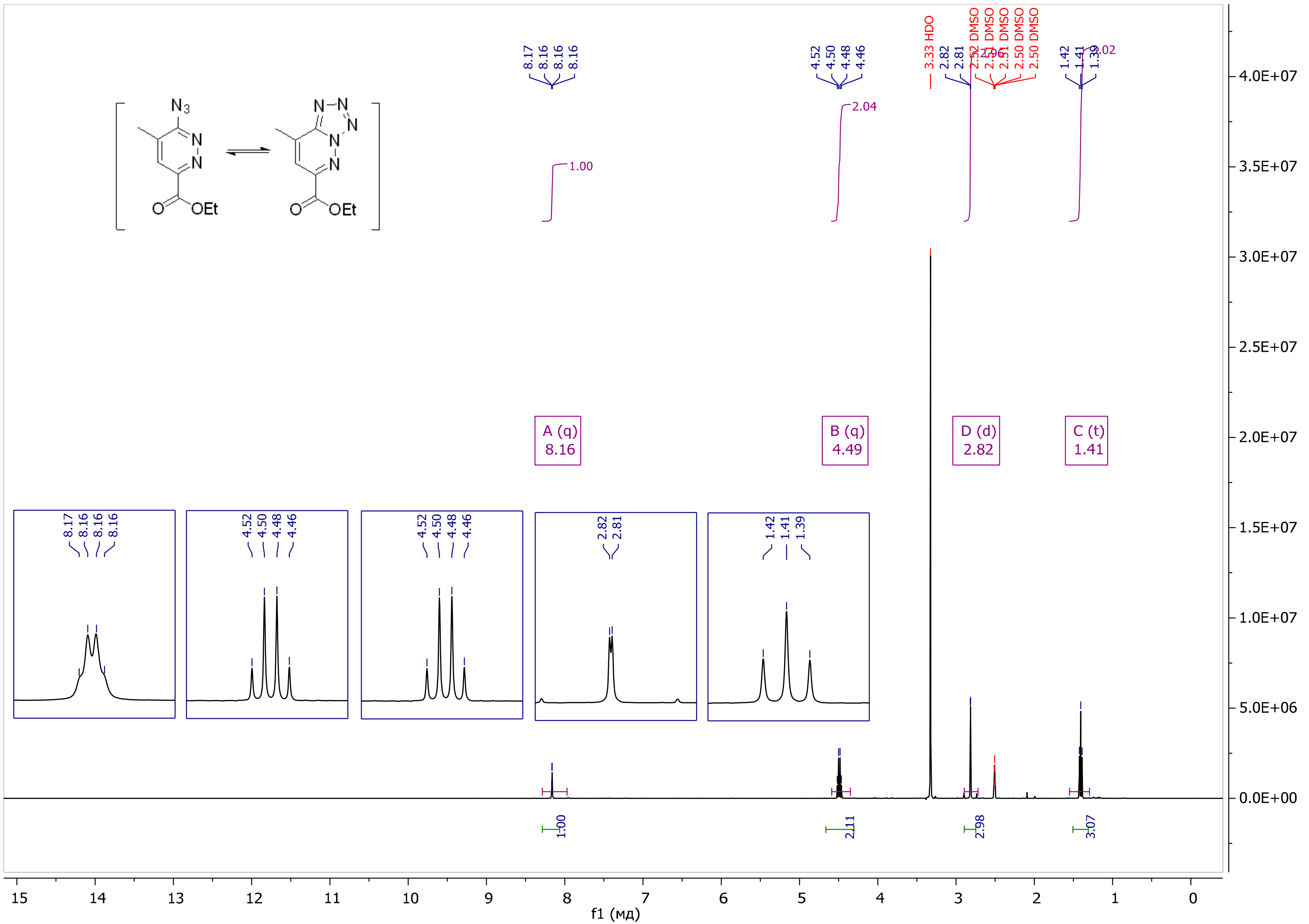

^{13}C NMR spectrum of ethyl 6-azido-5-methylpyridazine-3-carboxylate **20**

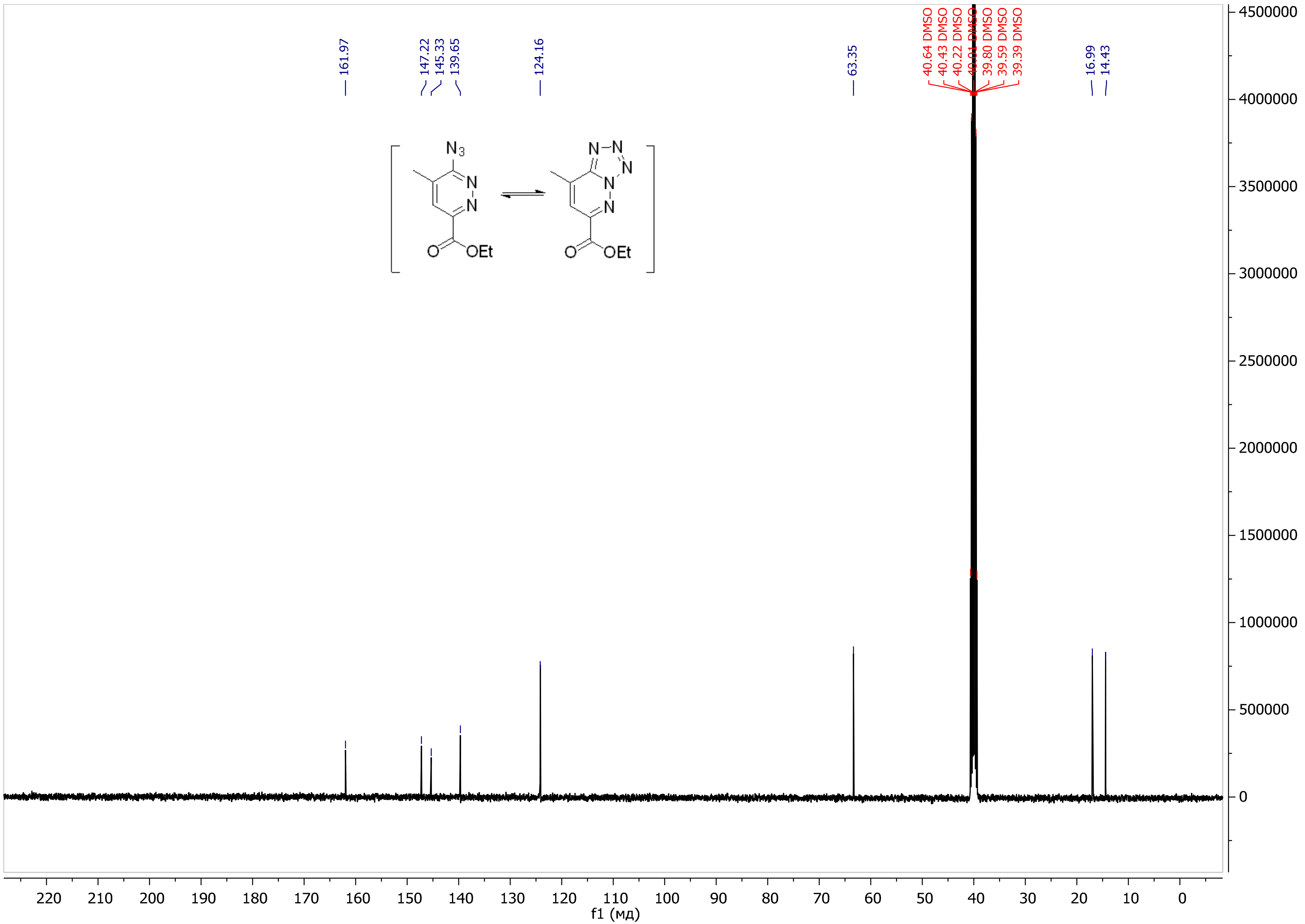

^{1}H NMR spectrum of ethyl 8-methyl-5,6-dihydrotetrazolo[1,5-b]pyridazine-6-carboxylate **20a**

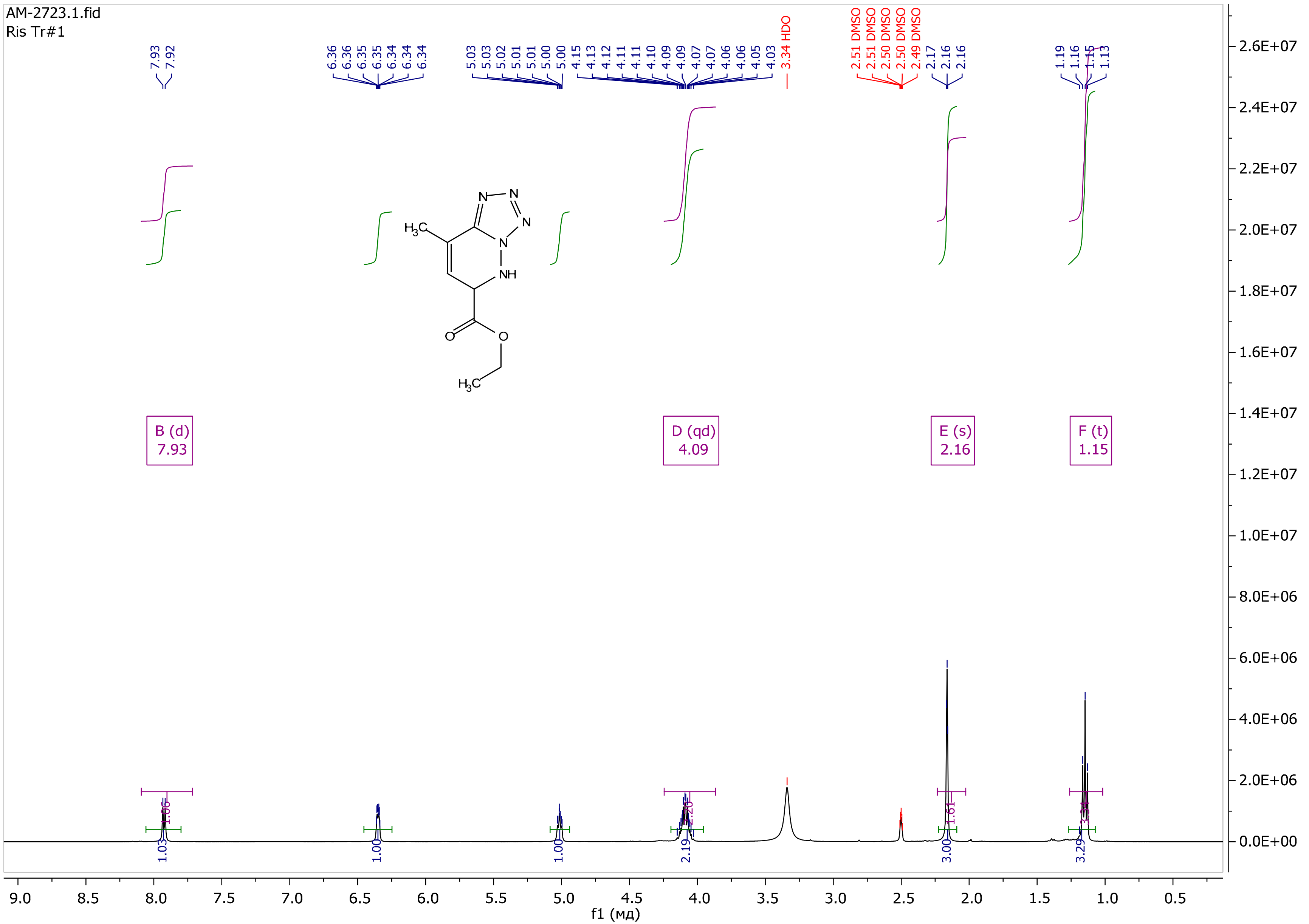

^{13}C NMR spectrum of ethyl 8-methyl-5,6-dihydrotetrazolo[1,5-b]pyridazine-6-carboxylate **20a**

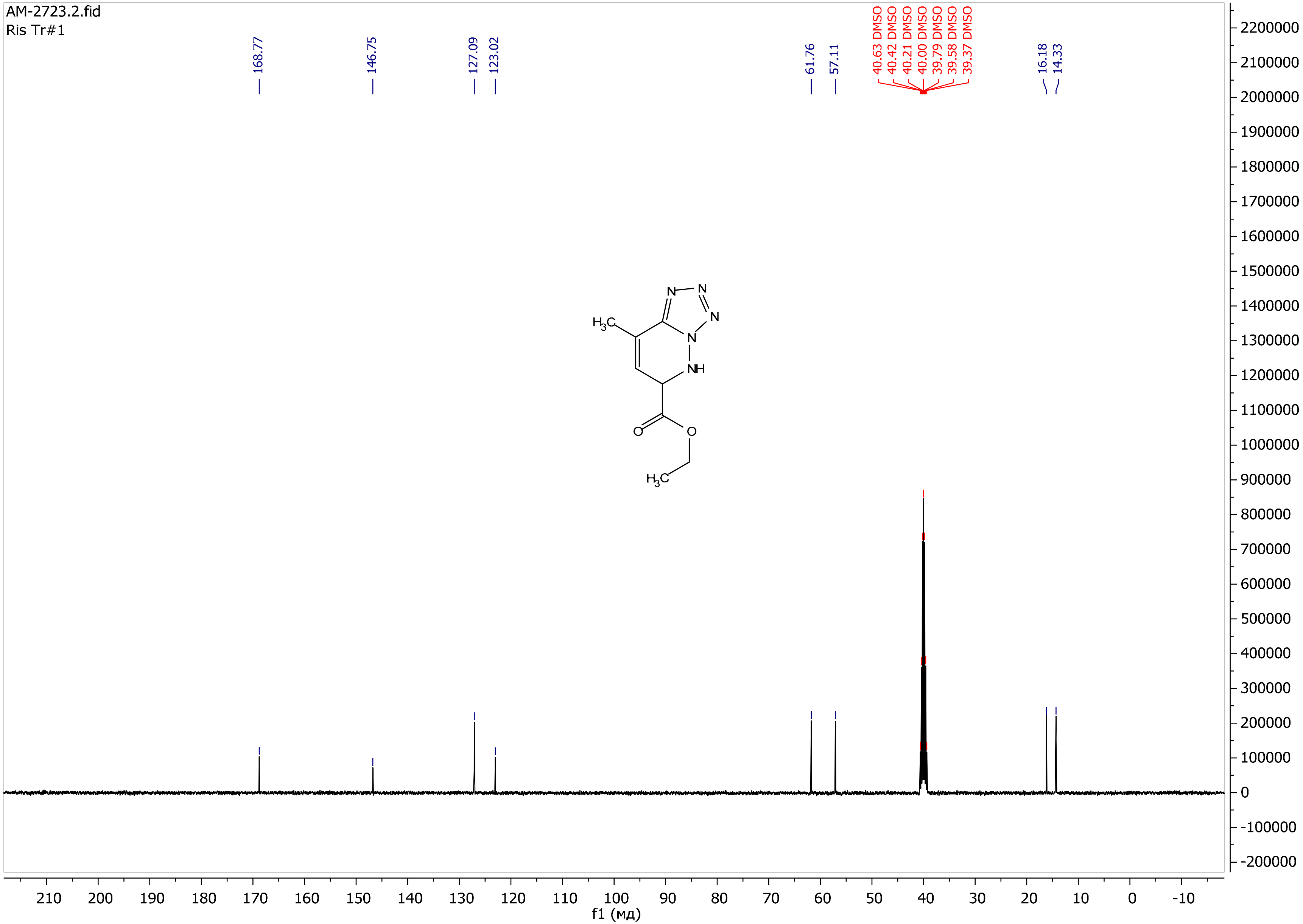

^{1}H NMR spectrum of ethyl 6-amino-5-methylpyridazine-3-carboxylate **21**

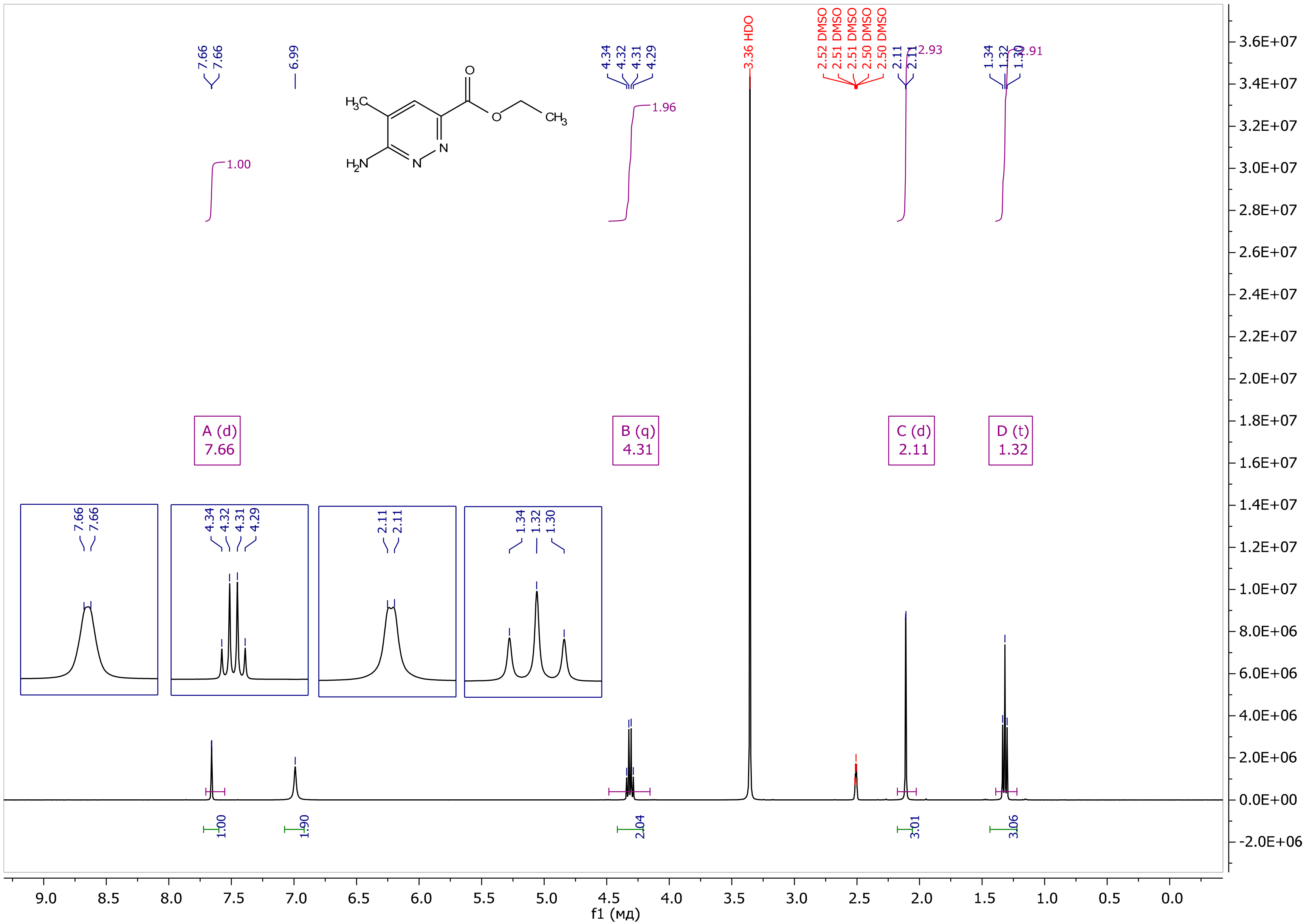

^{13}C NMR spectrum of ethyl 6-amino-5-methylpyridazine-3-carboxylate **21**

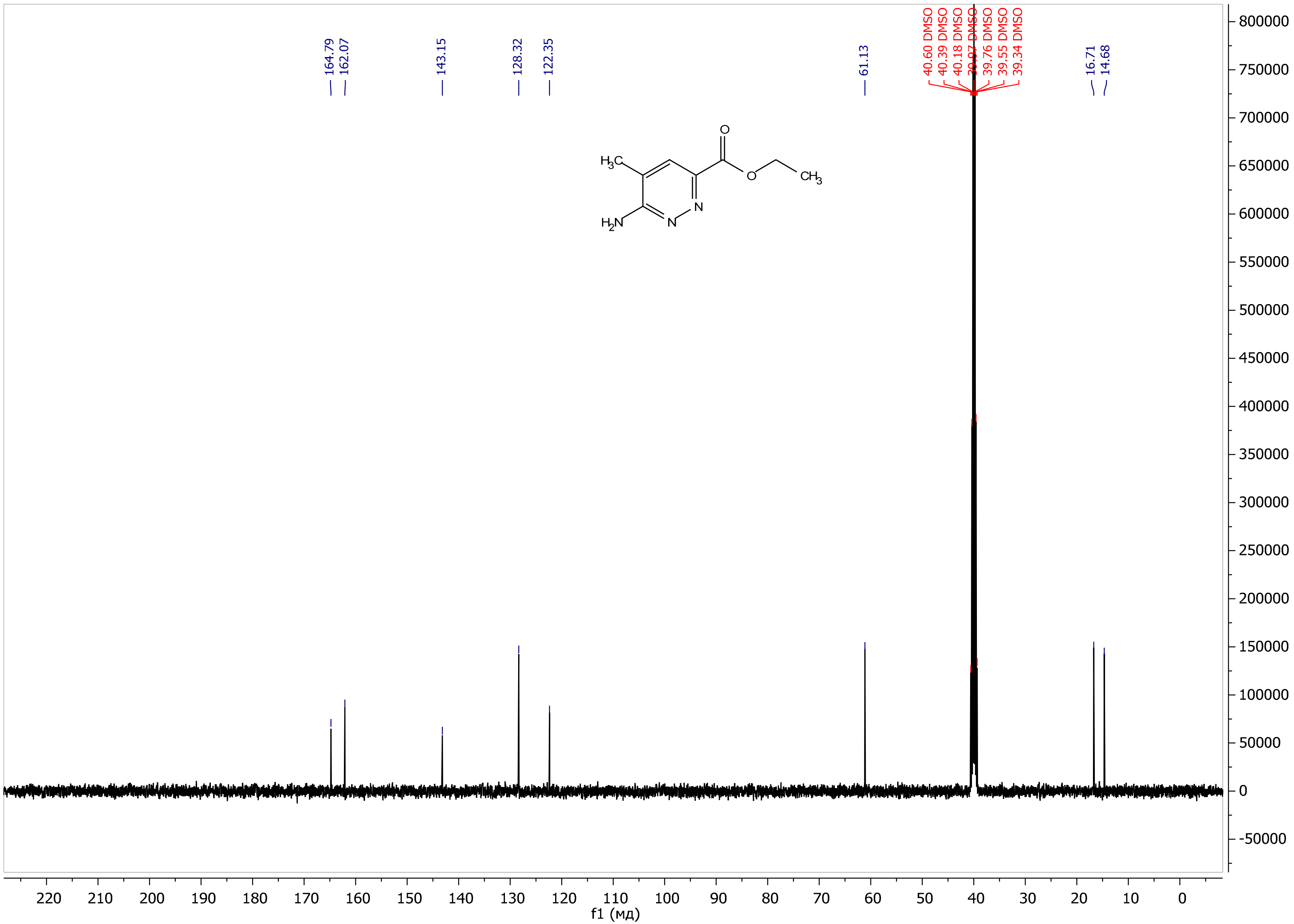

^{1}H NMR spectrum of ethyl 2,8-dimethylimidazo[1,2-b]pyridazine-6-carboxylate **22**

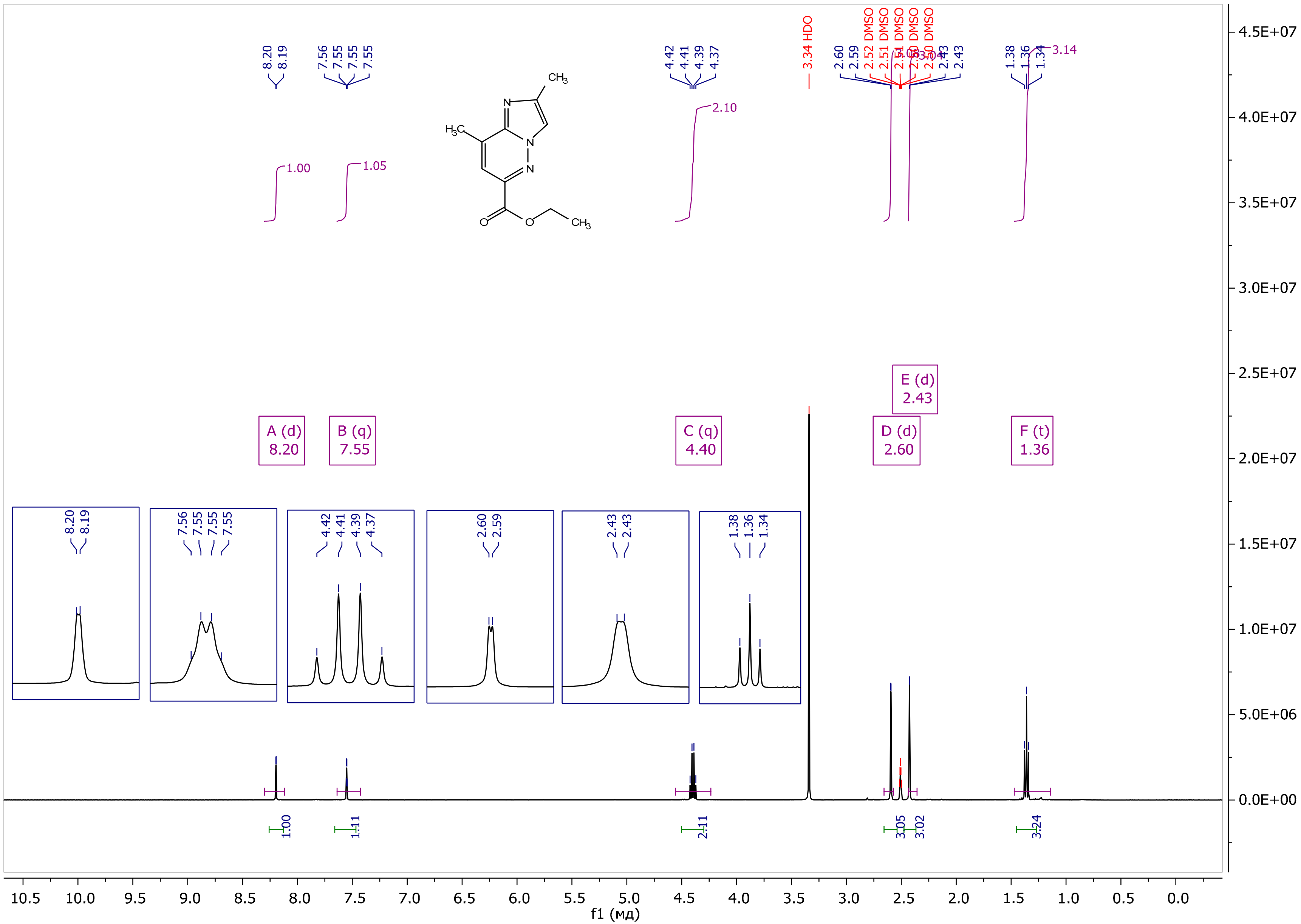

^{13}C NMR spectrum of ethyl 2,8-dimethylimidazo[1,2-b]pyridazine-6-carboxylate **22**

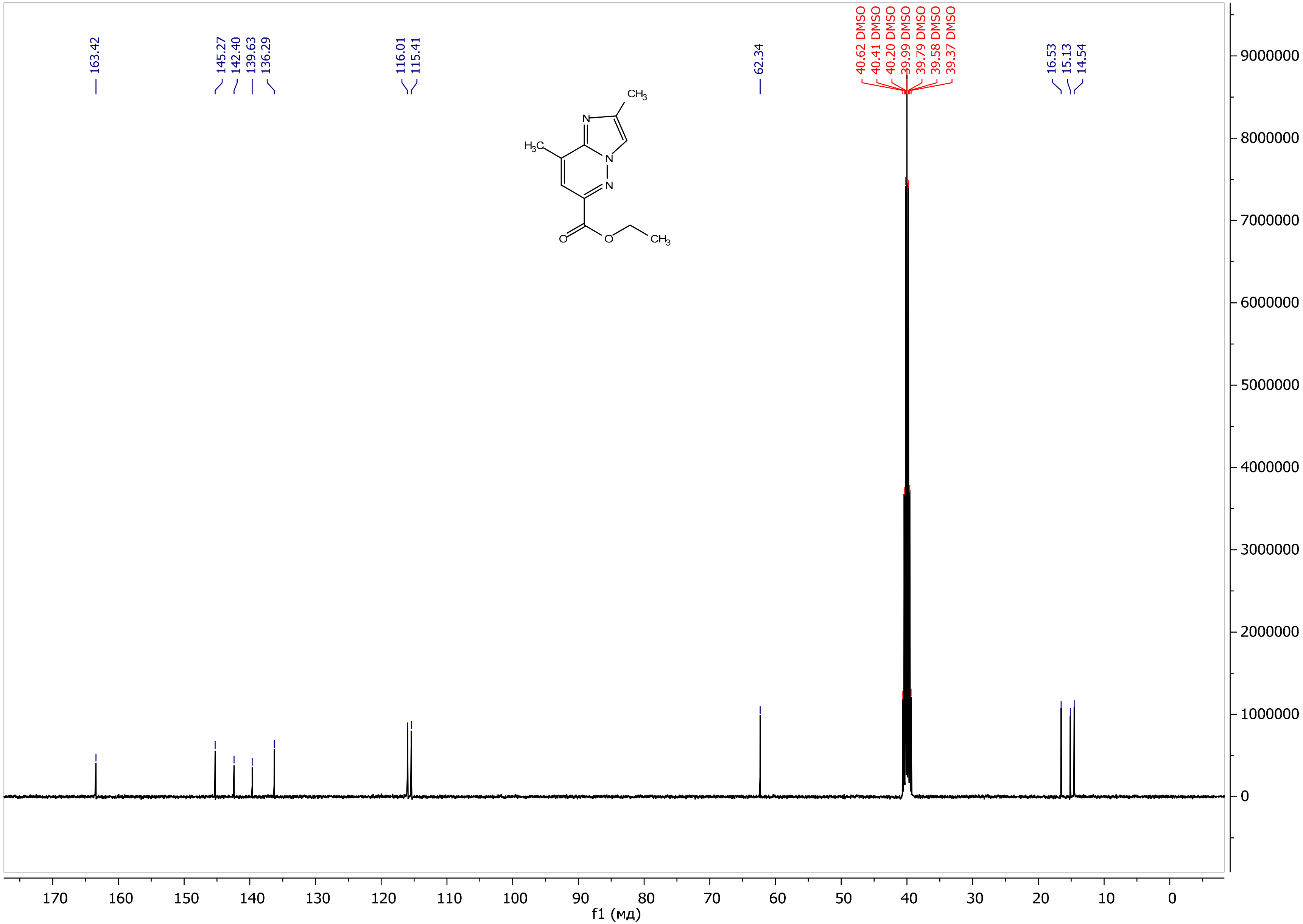

¹H NMR spectrum of 2,8-dimethylimidazo[1,2-b]pyridazine-6-carboxylic acid **13**

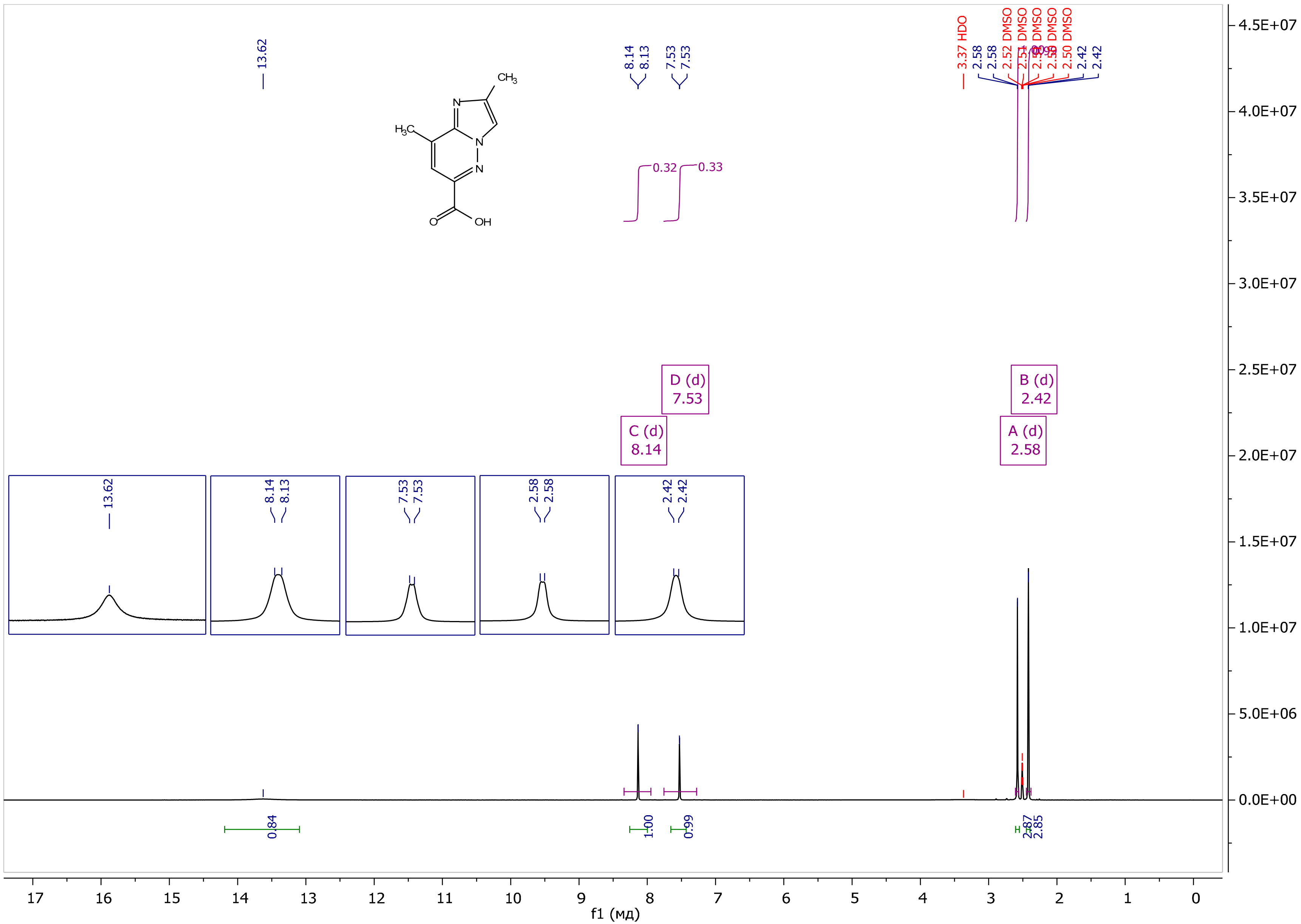

^{13}C NMR spectrum of 2,8-dimethylimidazo[1,2-b]pyridazine-6-carboxylic acid **13**

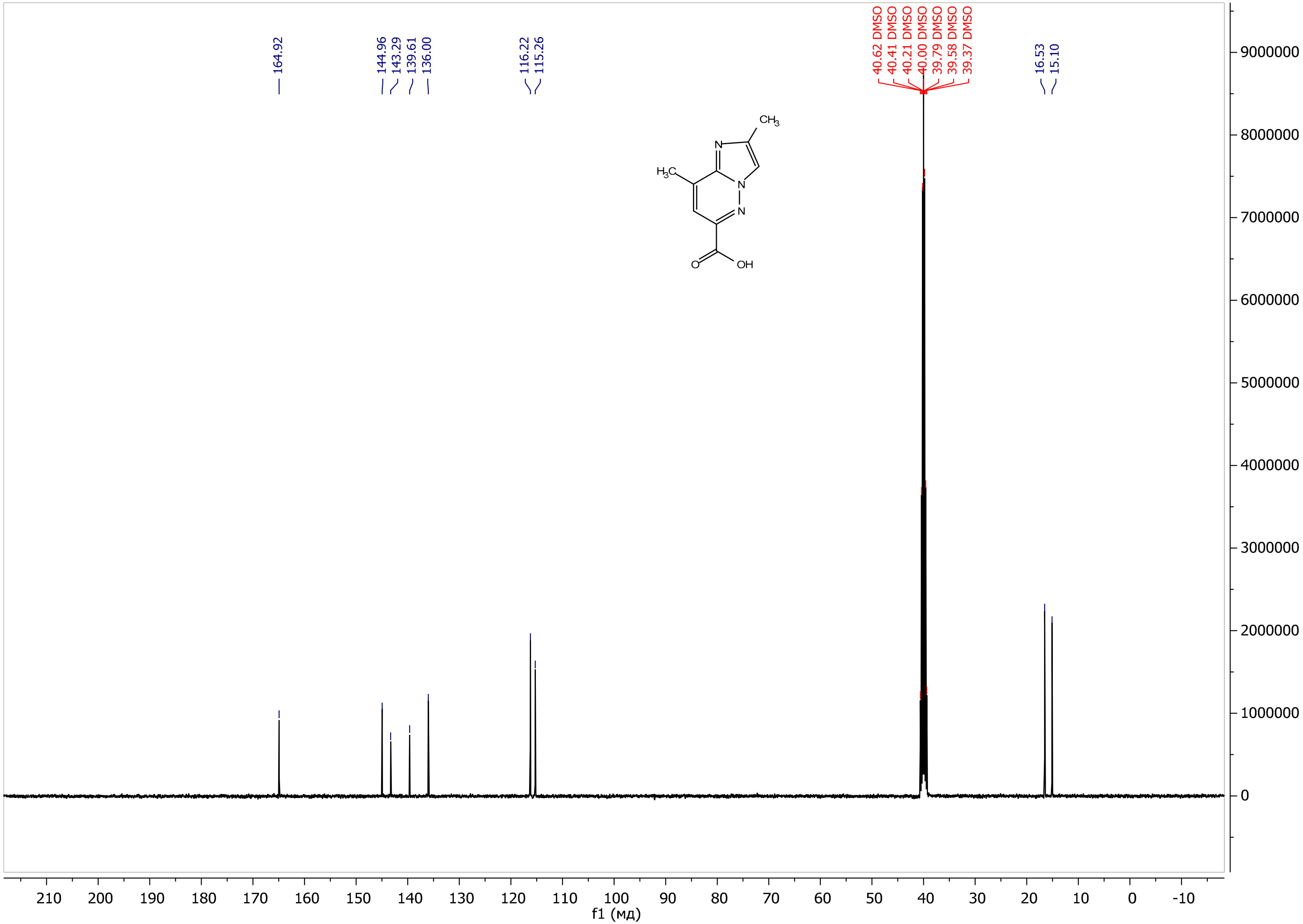

^{1}H NMR spectrum of tert-butyl 7-(6-(((2,2-dimethyl-4,6-dioxo-1,3-dioxan-5-ylidene)(2,8-dimethylimidazo[1,2-b]pyridazin-6-yl)methyl)amino)pyridin-3-yl)-4,7-diazaspiro[2.5]octane-4-carboxylate **26**

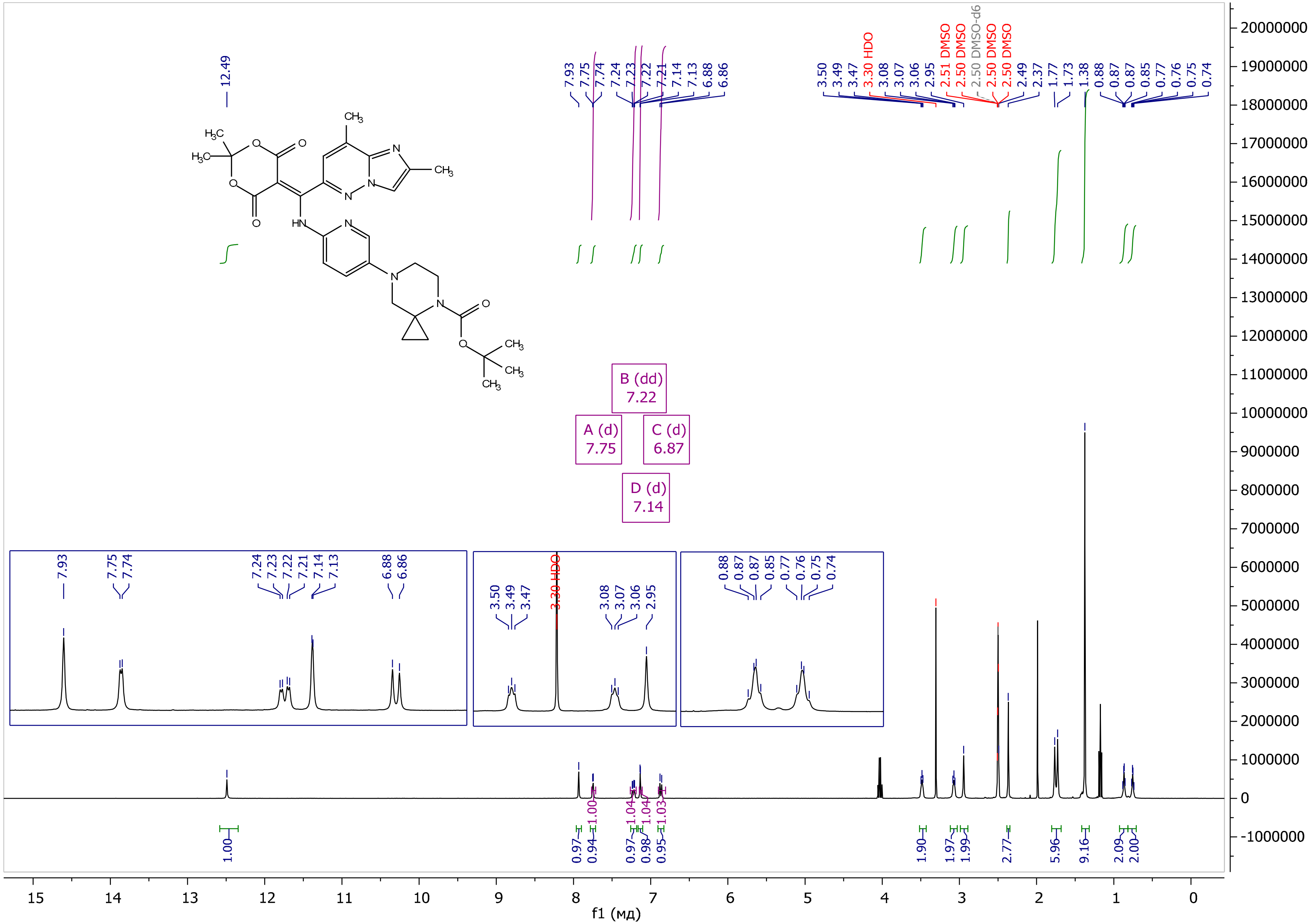

^{13}C NMR spectrum of tert-butyl 7-(6-(((2,2-dimethyl-4,6-dioxo-1,3-dioxan-5-ylidene)(2,8-dimethylimidazo[1,2-b]pyridazin-6-yl)methyl)amino)pyridin-3-yl)-4,7-diazaspiro[2.5]octane-4-carboxylate **26**

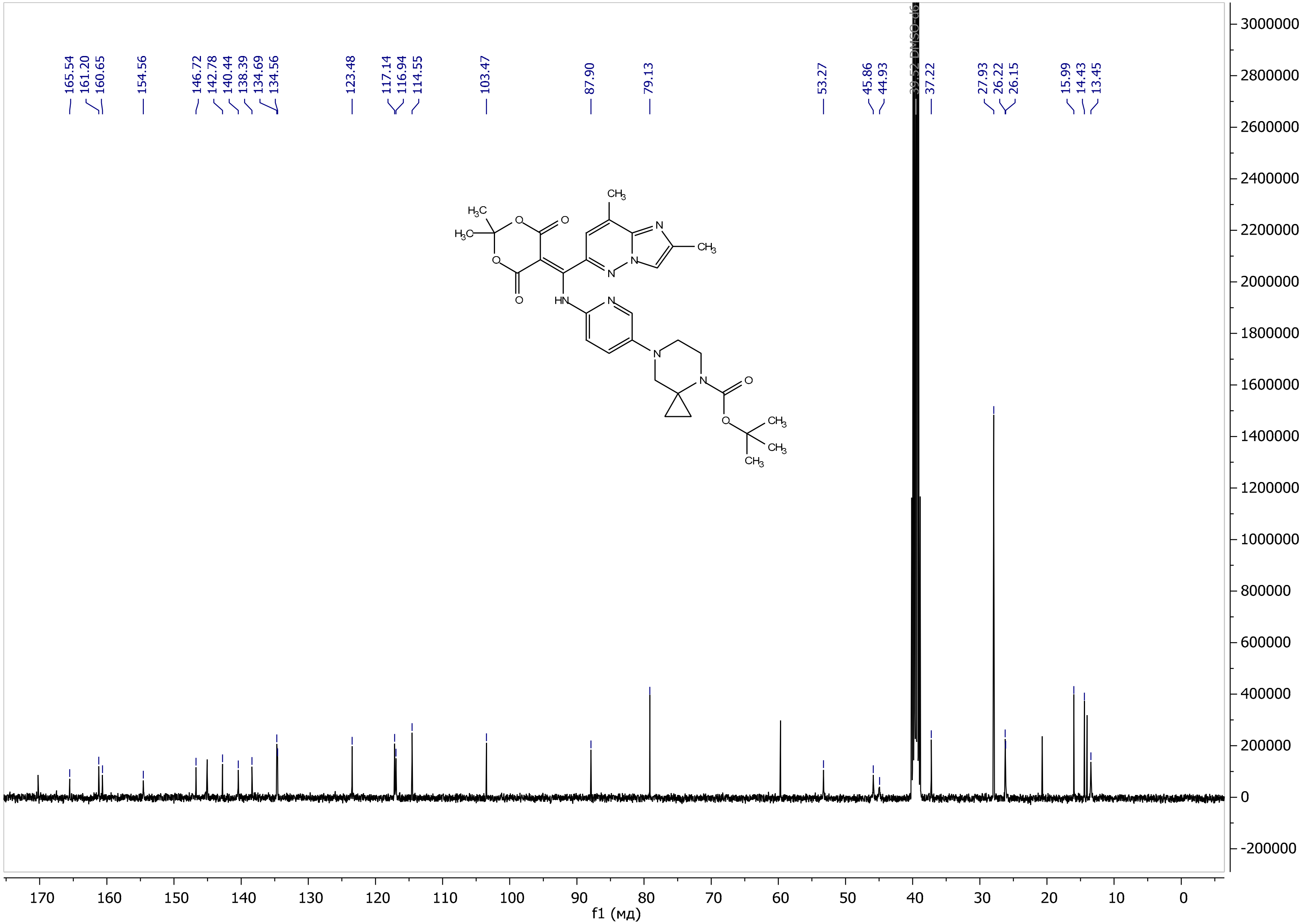

^{1}H NMR spectrum of 7-(4-(tert-butoxycarbonyl)-4,7-diazaspiro[2.5]octan-7-yl)-2-(2,8-dimethylimidazo[1,2-b]pyridazin-6-yl)-4-oxo-4H-pyrido[1,2-a]pyrimidine-3-carboxylic acid **27**

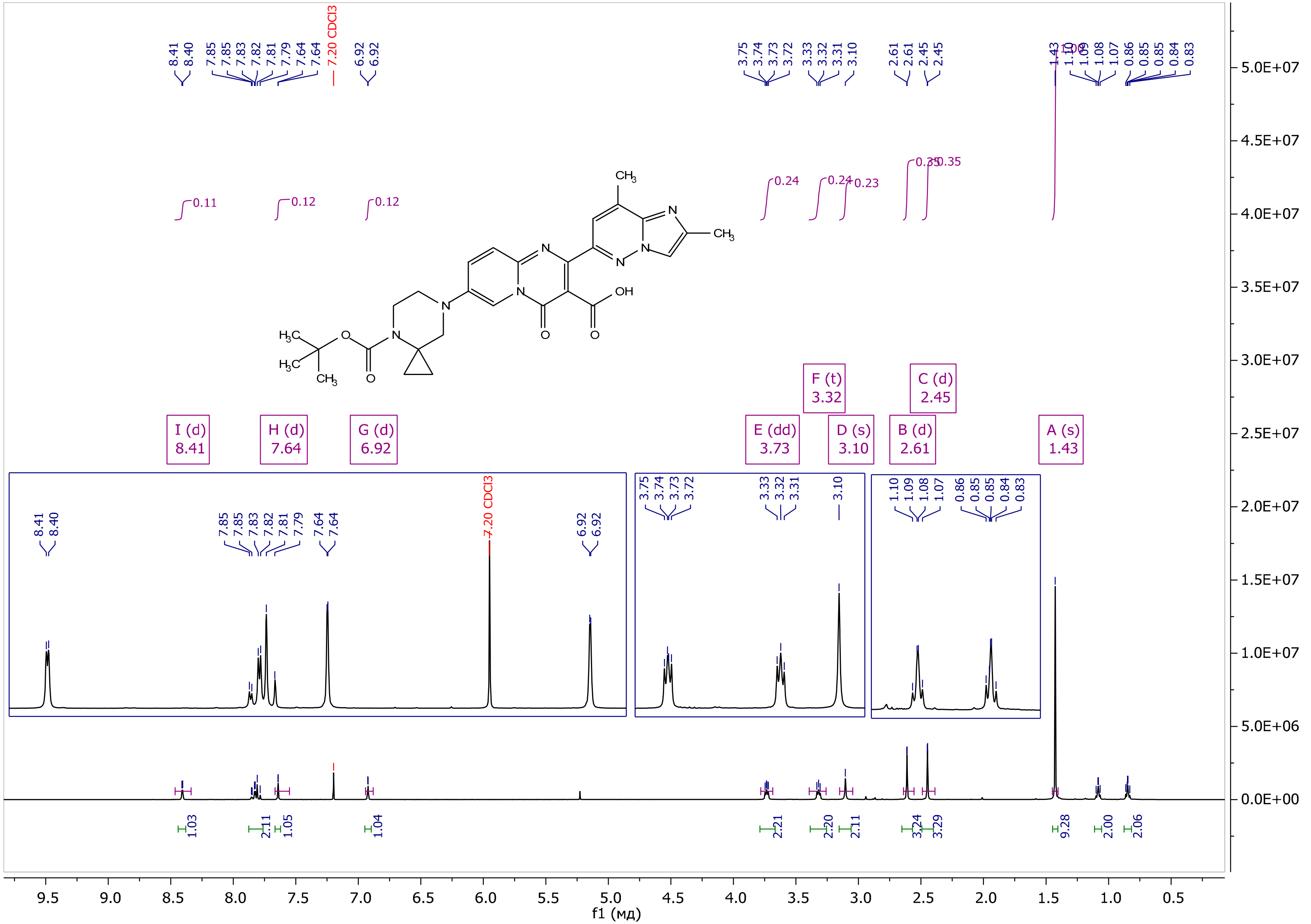

^{13}C NMR spectrum of 7-(4-(tert-butoxycarbonyl)-4,7-diazaspiro[2.5]octan-7-yl)-2-(2,8-dimethylimidazo[1,2-b]pyridazin-6-yl)-4-oxo-4H-pyrido[1,2-a]pyrimidine-3-carboxylic acid **27**

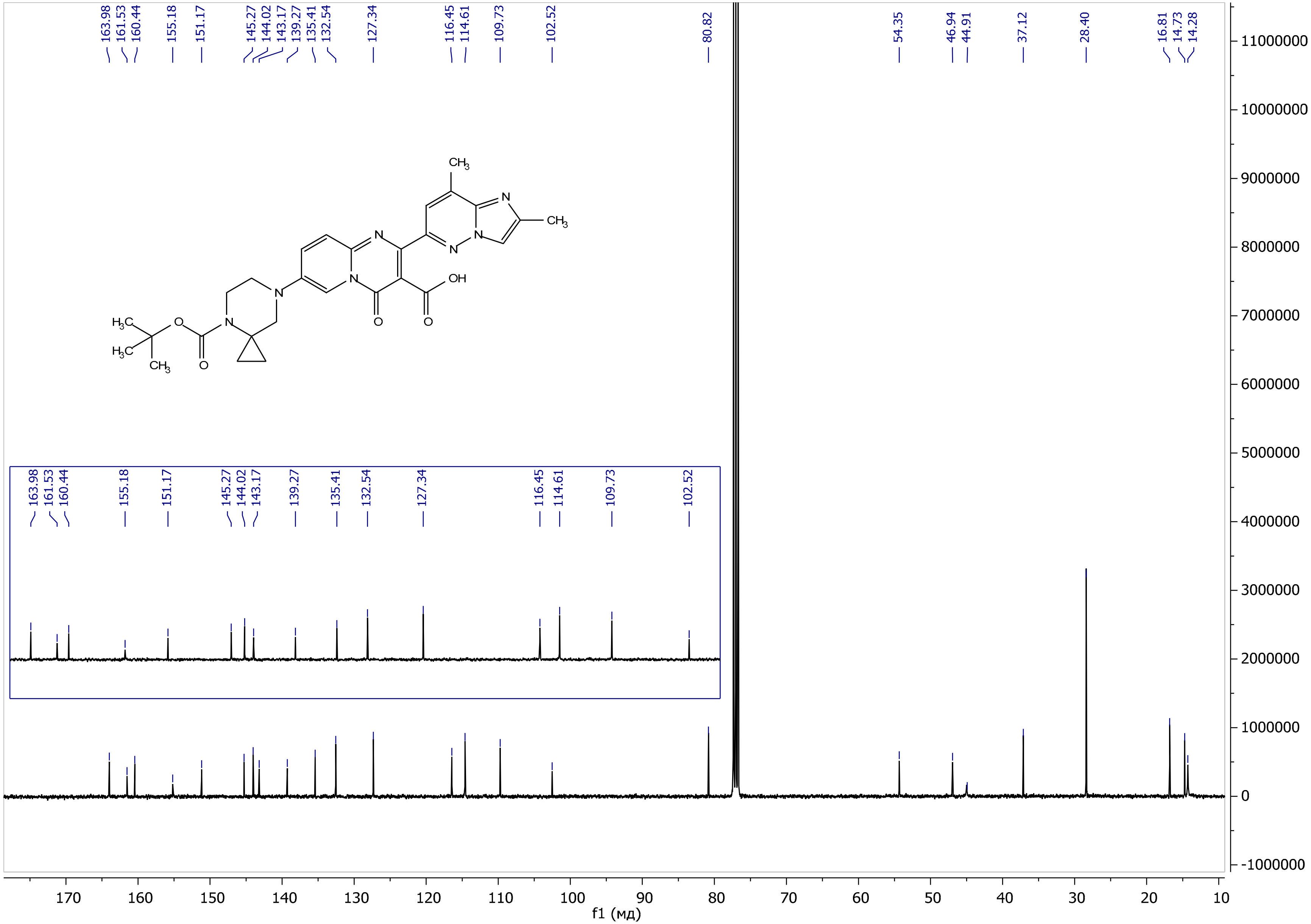

^{1}H NMR spectrum of 2-(2,8-dimethylimidazo[1,2-b]pyridazin-6-yl)-7-(4,7-diazaspiro[2.5]octan-7-yl)-4H-pyrido[1,2-a]pyrimidin-4-one (Risdiplam)

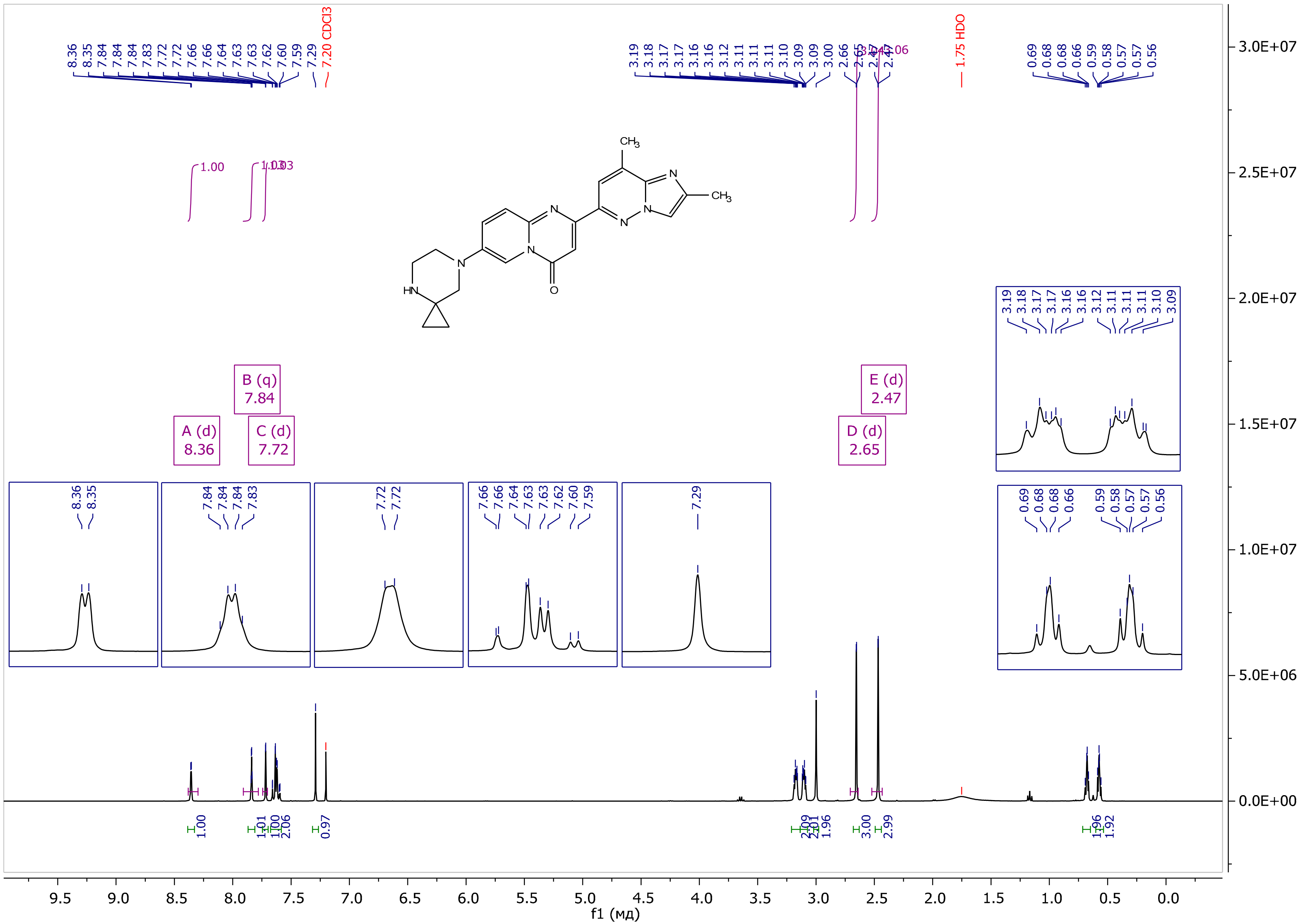

^{13}C NMR spectrum of 2-(2,8-dimethylimidazo[1,2-b]pyridazin-6-yl)-7-(4,7-diazaspiro[2.5]octan-7-yl)-4H-pyrido[1,2-a]pyrimidin-4-one (Risdiplam)

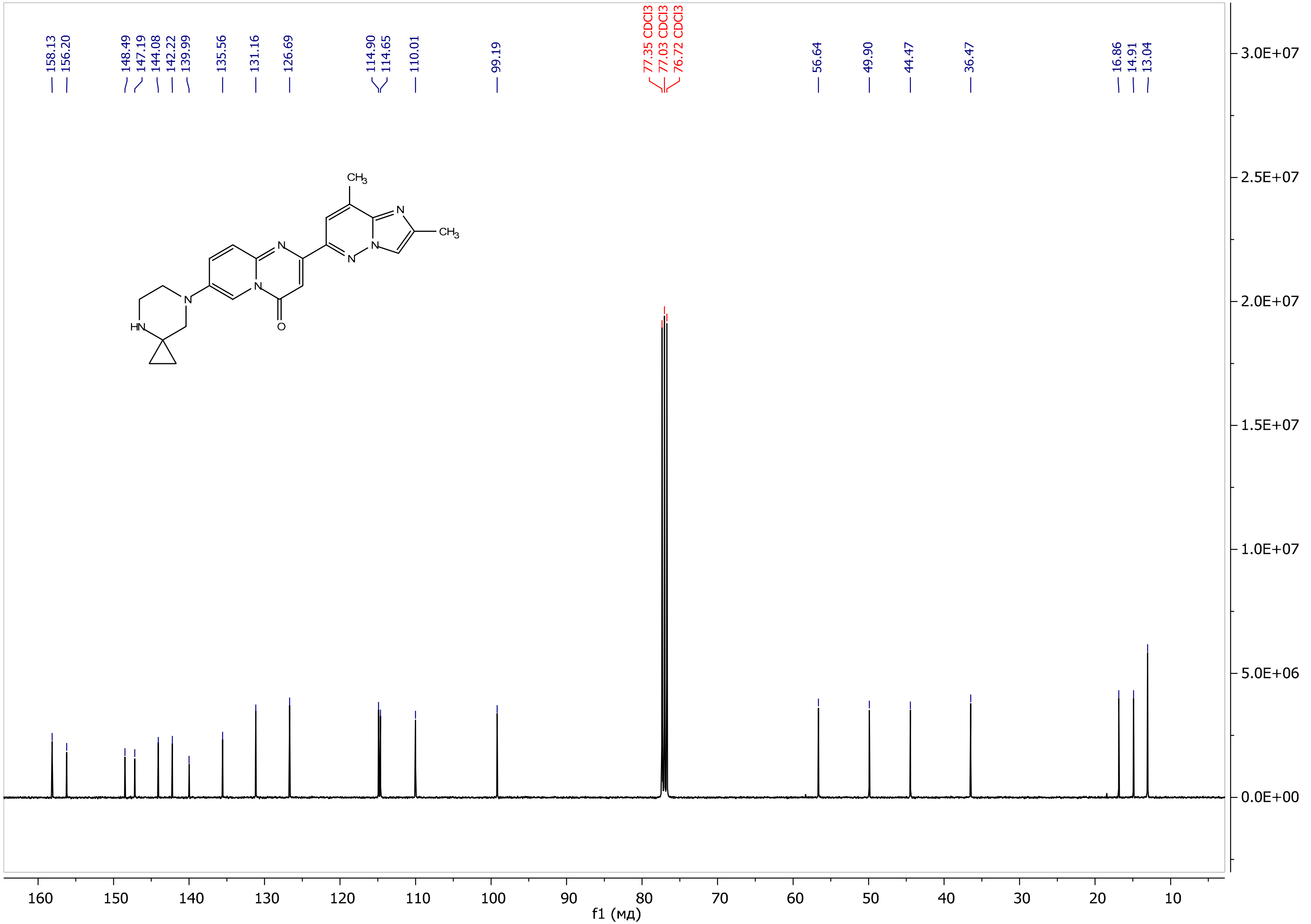

^{1}H NMR spectrum of tert-butyl 4,7-diazaspiro[2.5]octane-4-carboxylate **30**

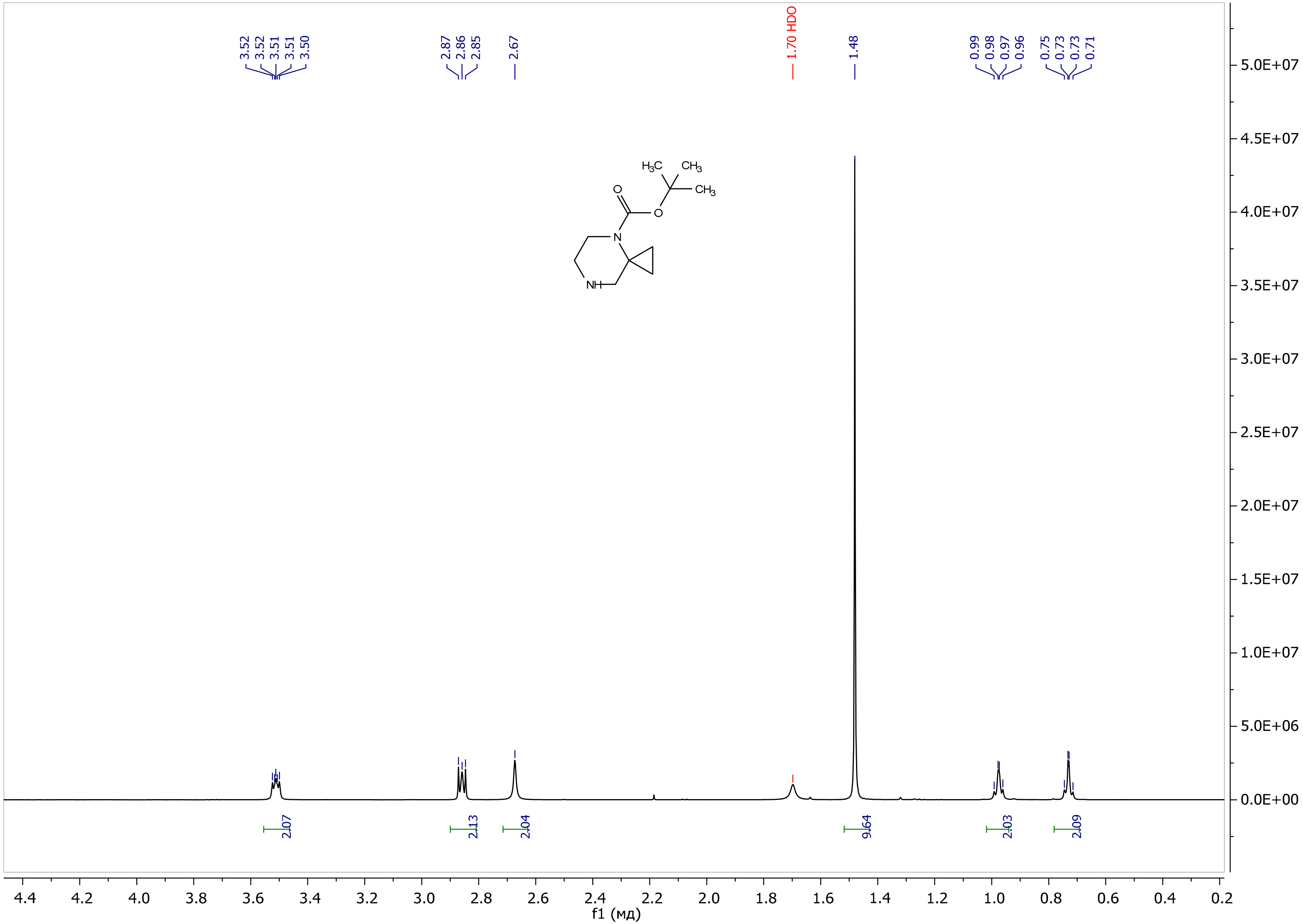

^{13}C NMR spectrum of tert-butyl 4,7-diazaspiro[2.5]octane-4-carboxylate **30**

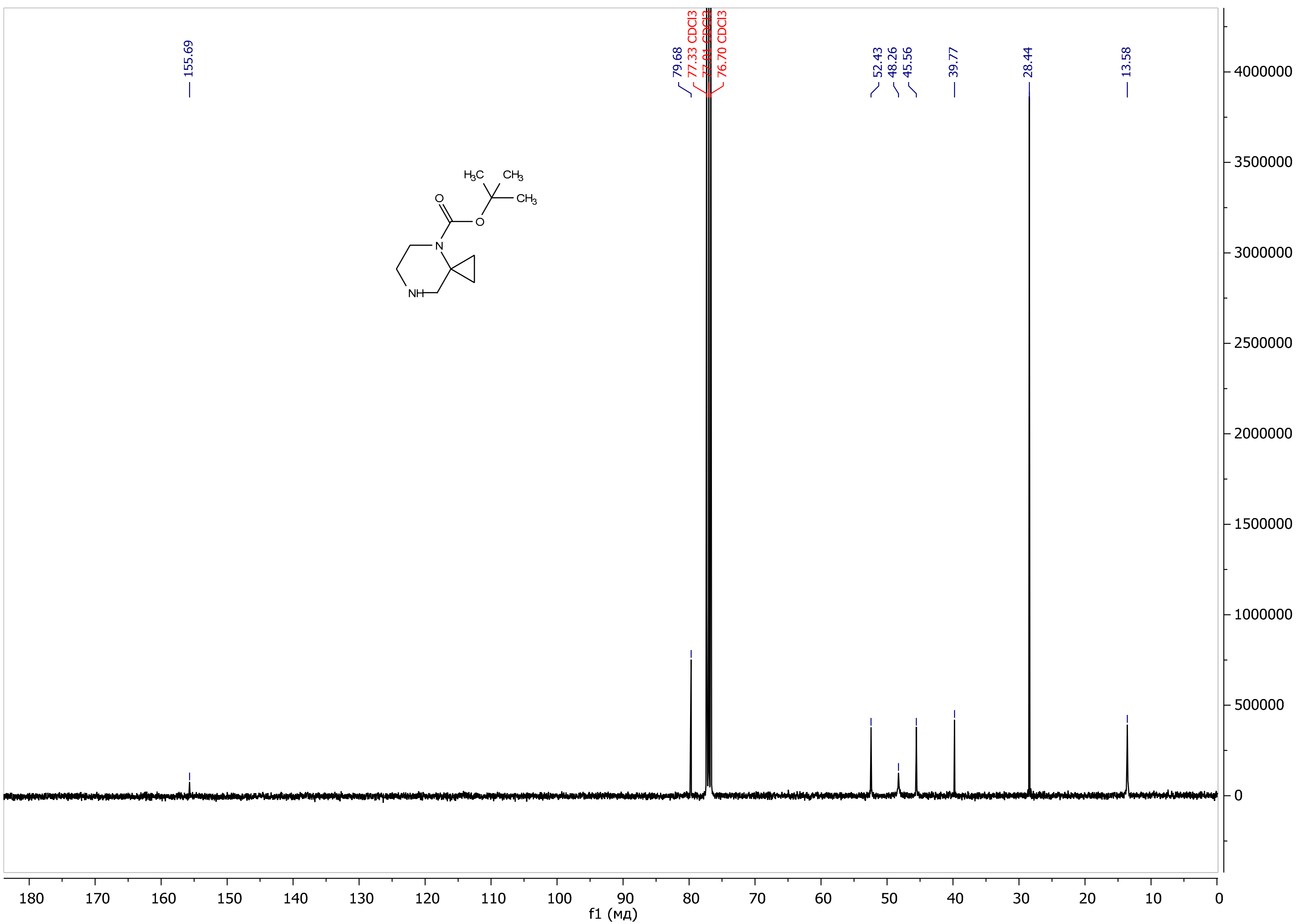

^{1}H NMR spectrum of tert-butyl 7-(6-nitropyridin-3-yl)-4,7-diazaspiro[2.5]octane-4-carboxylate **31**

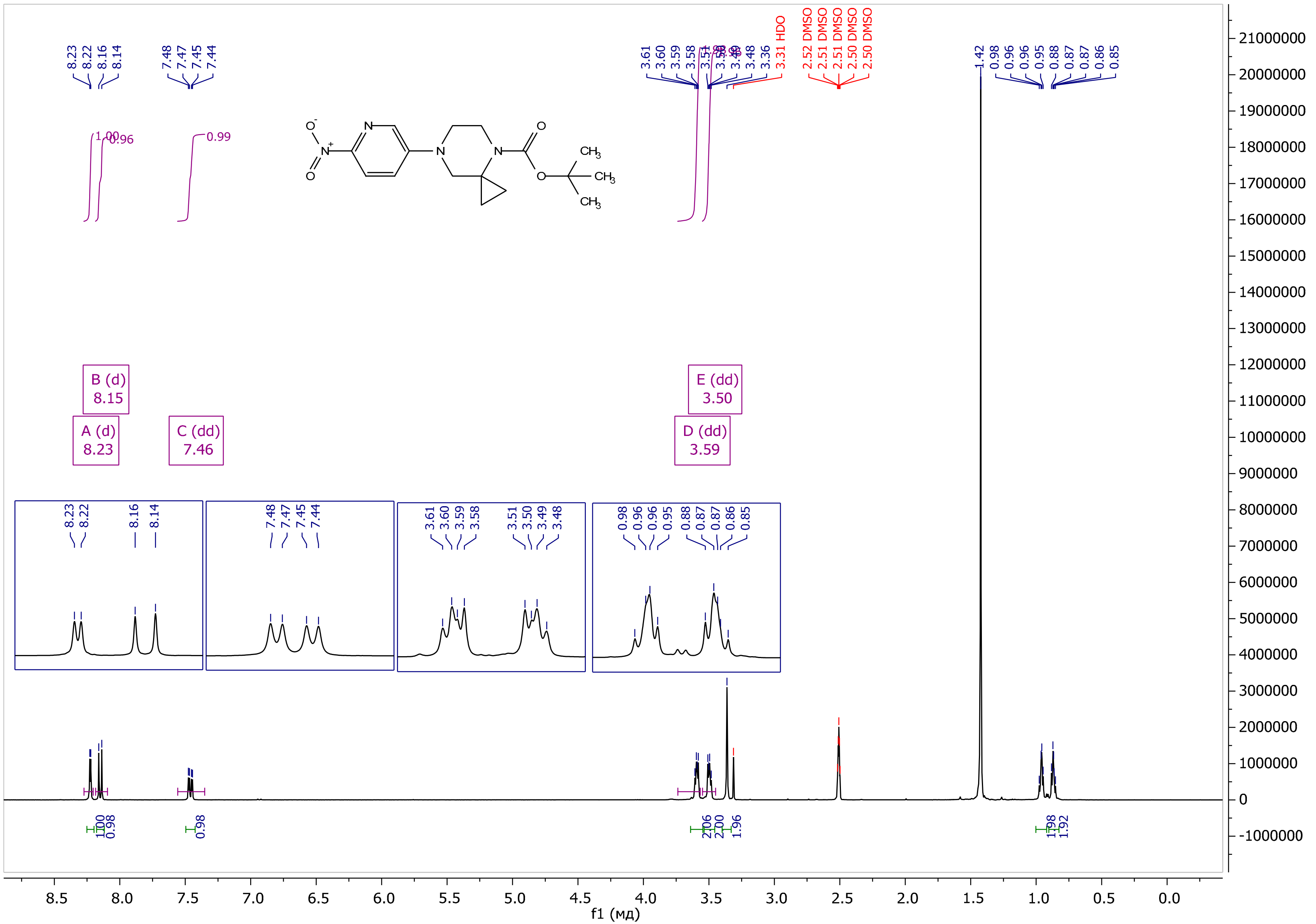

^{13}C NMR spectrum of tert-butyl 7-(6-nitropyridin-3-yl)-4,7-diazaspiro[2.5]octane-4-carboxylate **31**

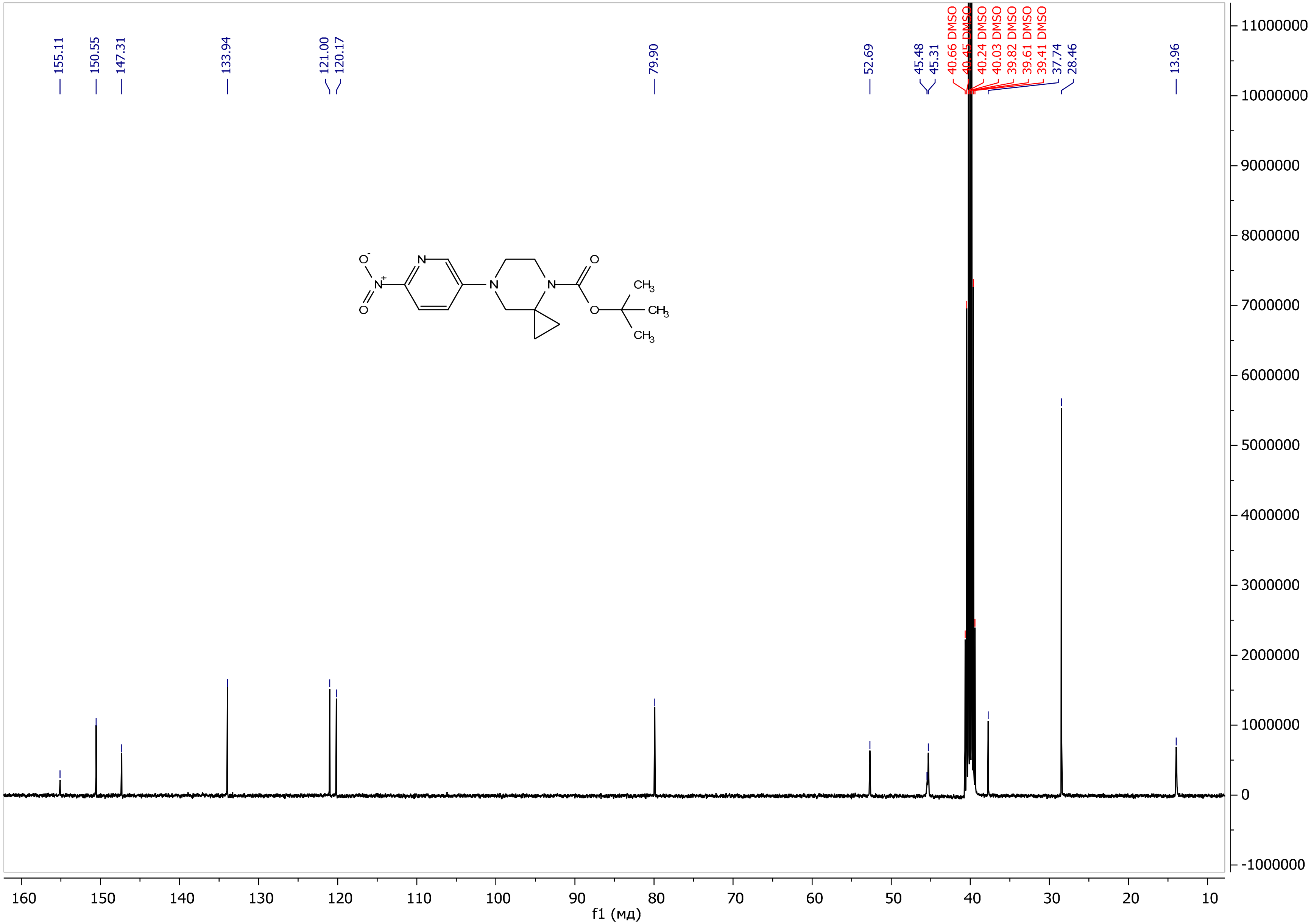

^{1}H NMR spectrum of tert-butyl 7-(6-aminopyridin-3-yl)-4,7-diazaspiro[2.5]octane-4-carboxylate **28**

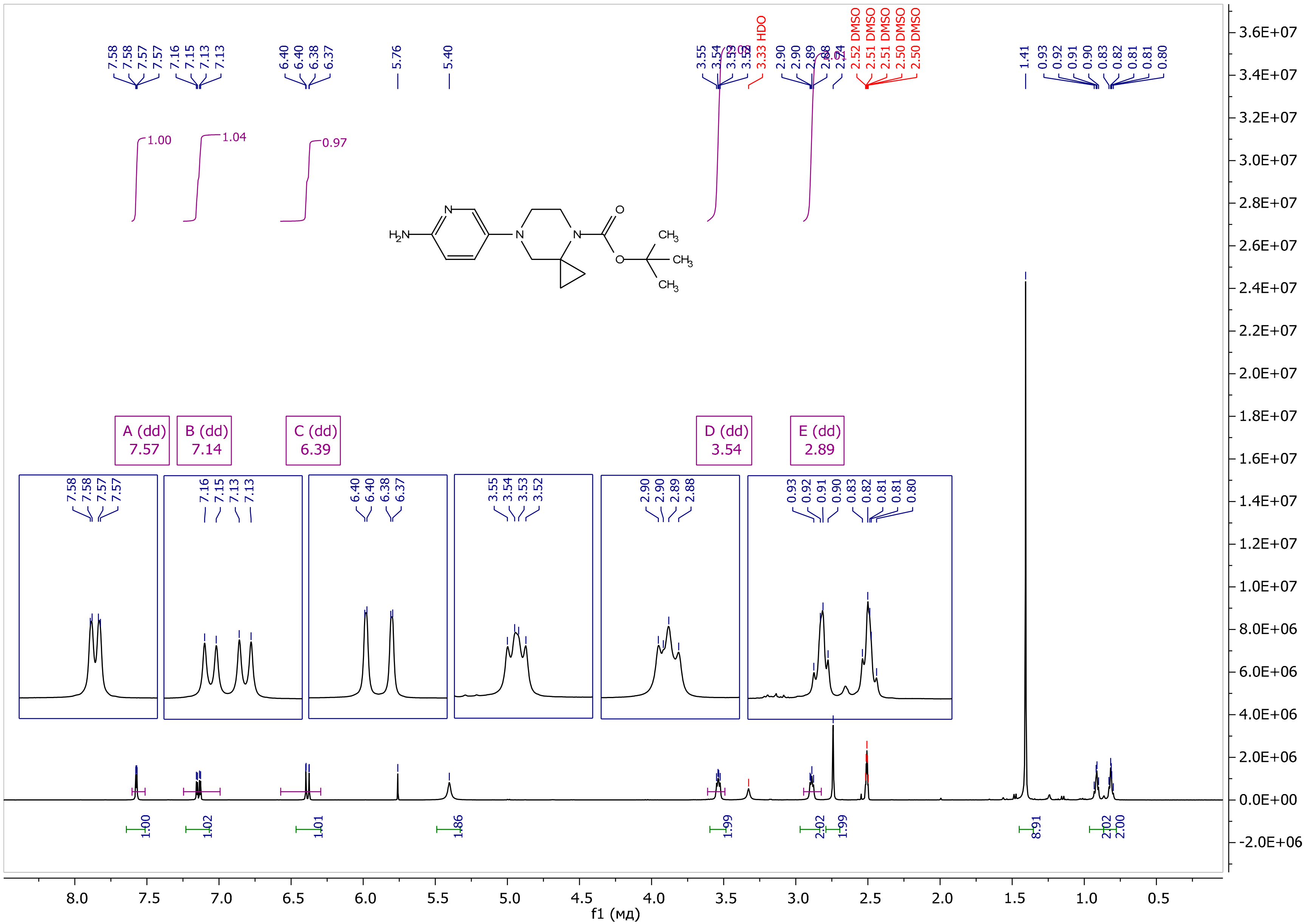

^{13}C NMR spectrum of tert-butyl 7-(6-aminopyridin-3-yl)-4,7-diazaspiro[2.5]octane-4-carboxylate **28**

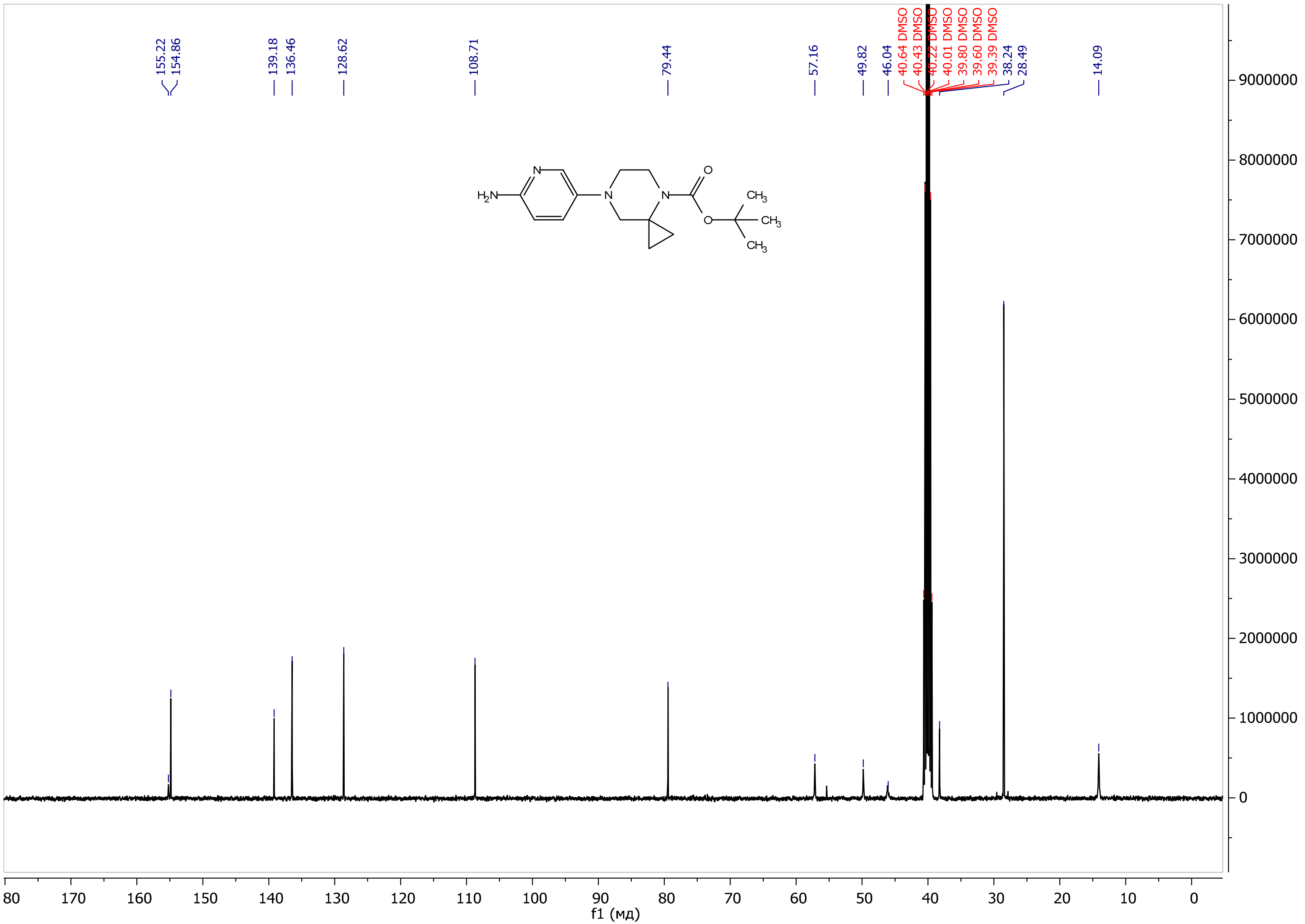

Copies of HRMS spectra

<table>
<tr><th>Compound</th><th>Structure</th><th>HRMS spectra (MS and MS2, correspondingly)</th></tr>
<tr><td>13</td><td></td><td>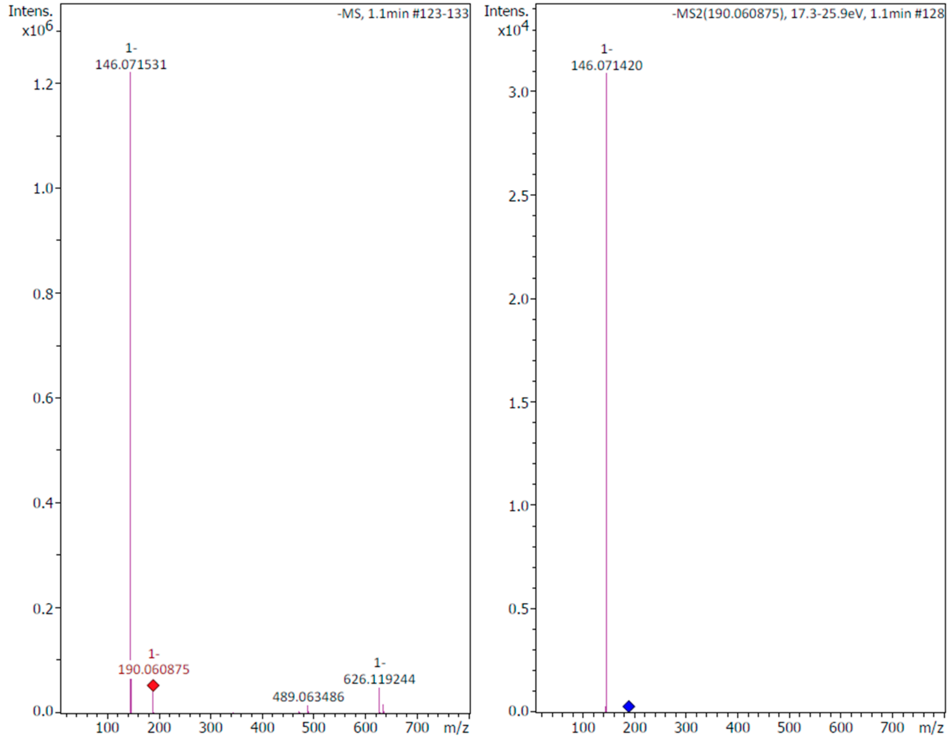
</td></tr>
<tr><td>17</td><td></td><td>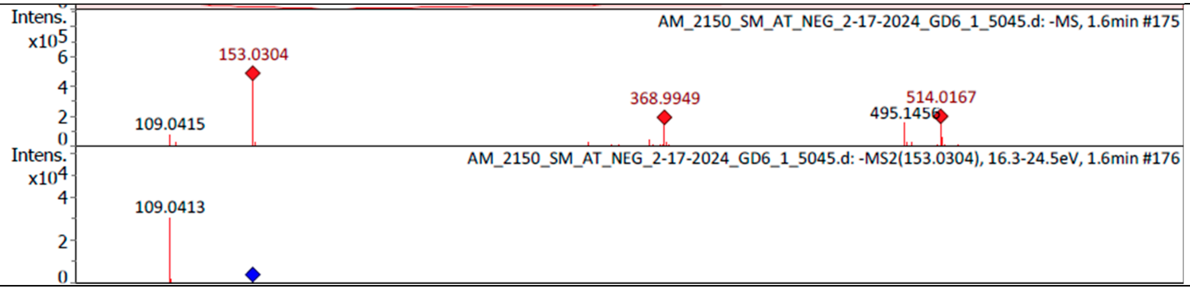
</td></tr>
</table>

**18**			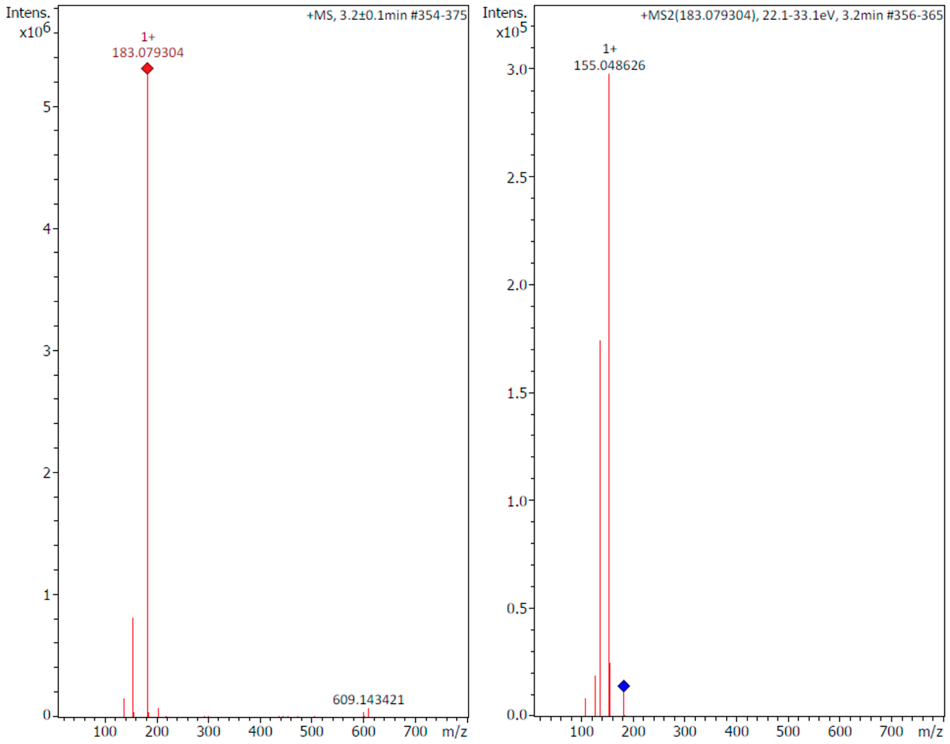Intens. x10^6<br>+MS, 3.2±0.1min #354-375<br>1+<br>183.079304<br>609.143421<br>m/z<br>Intens. x10^5<br>+MS2(183.079304), 22.1-33.1eV, 3.2min #356-365<br>1+<br>155.048626<br>m/z

**19**		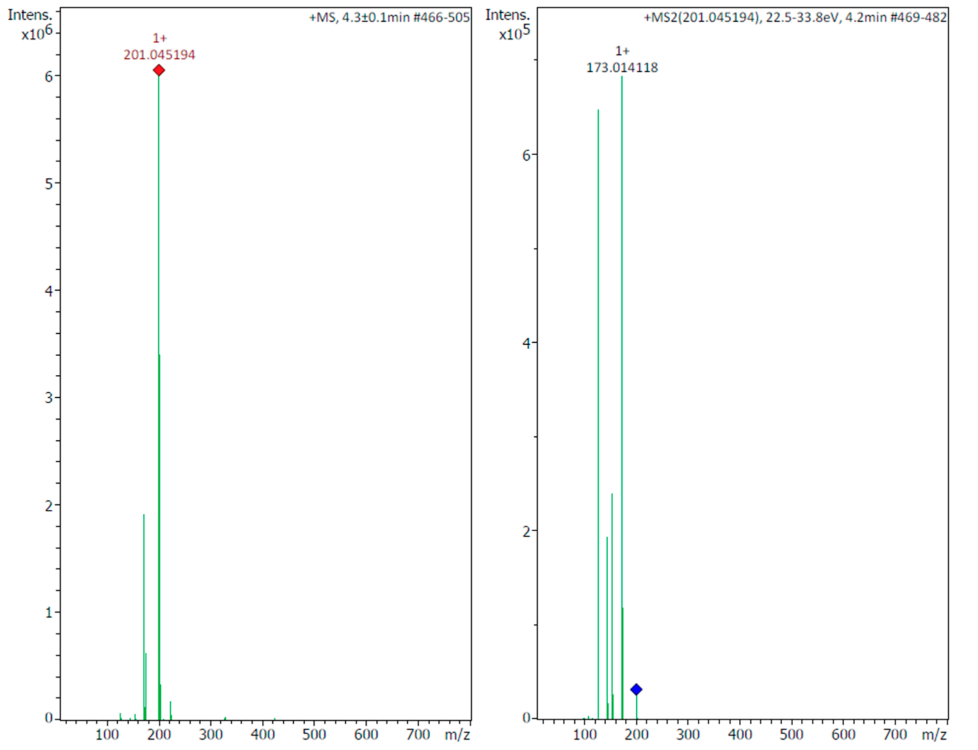Intens.<br>x10^6<br>+MS, 4.3±0.1min #466-505<br>1+<br>201.045194<br>m/z<br>Intens.<br>x10^5<br>+MS2(201.045194), 22.5-33.8eV, 4.2min #469-482<br>1+<br>173.014118<br>m/z

**20**			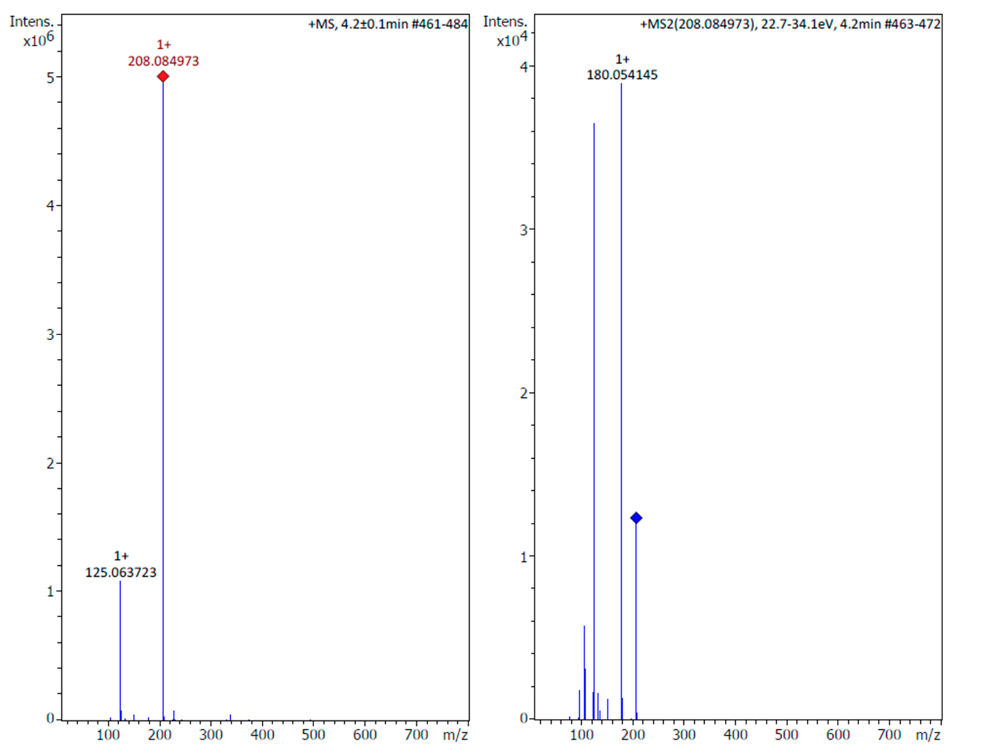Intens. x10^6<br>+MS, 4.2±0.1min #461-484<br>1+ 208.084973<br>1+ 125.063723<br>m/z<br>Intens. x10^4<br>+MS2(208.084973), 22.7-34.1eV, 4.2min #463-472<br>1+ 180.054145<br>m/z

**21**			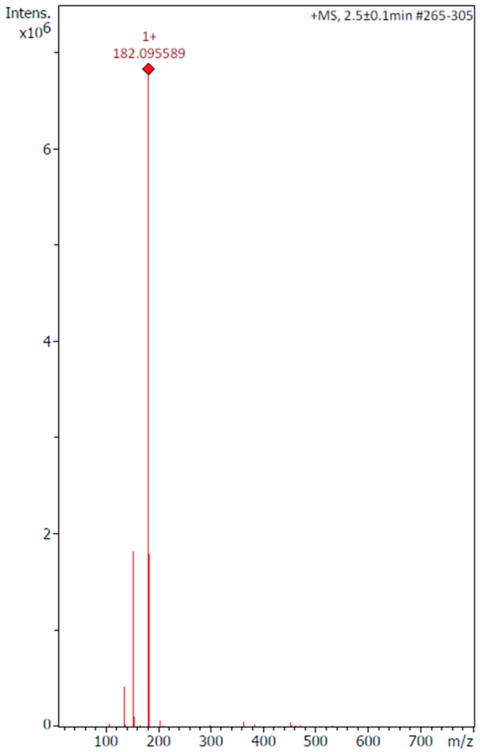Intens.<br>x10^6<br>+MS, 2.5±0.1min #265-305<br>1+<br>182.095589<br>m/z 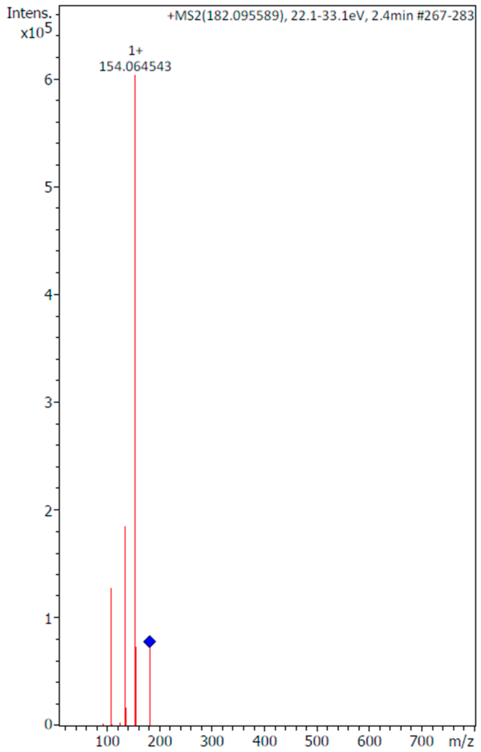Intens.<br>x10^5<br>+MS2(182.095589), 22.1-33.1eV, 2.4min #267-283<br>1+<br>154.064543<br>m/z

**22**			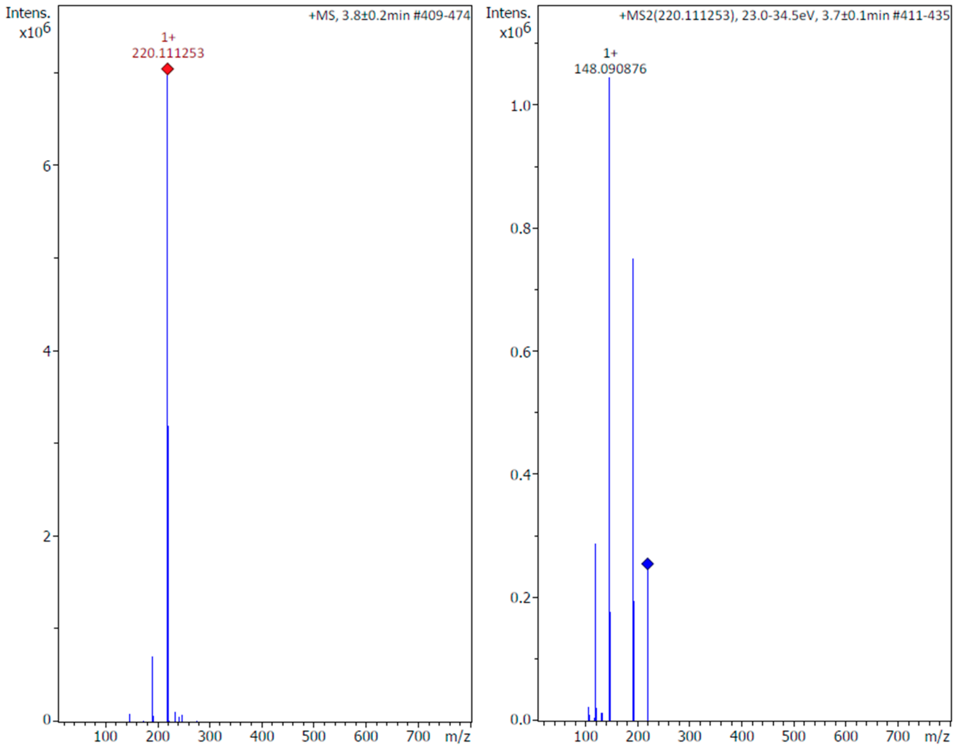

Intens.
x10^6
+MS, 3.8±0.2min #409-474
1+
220.111253
0
2
4
6
100
200
300
400
500
600
700
m/z
Intens.
x10^6
+MS2(220.111253), 23.0-34.5eV, 3.7±0.1min #411-435
1+
148.090876
0.0
0.2
0.4
0.6
0.8
1.0
100
200
300
400
500
600
700
m/z

**27**			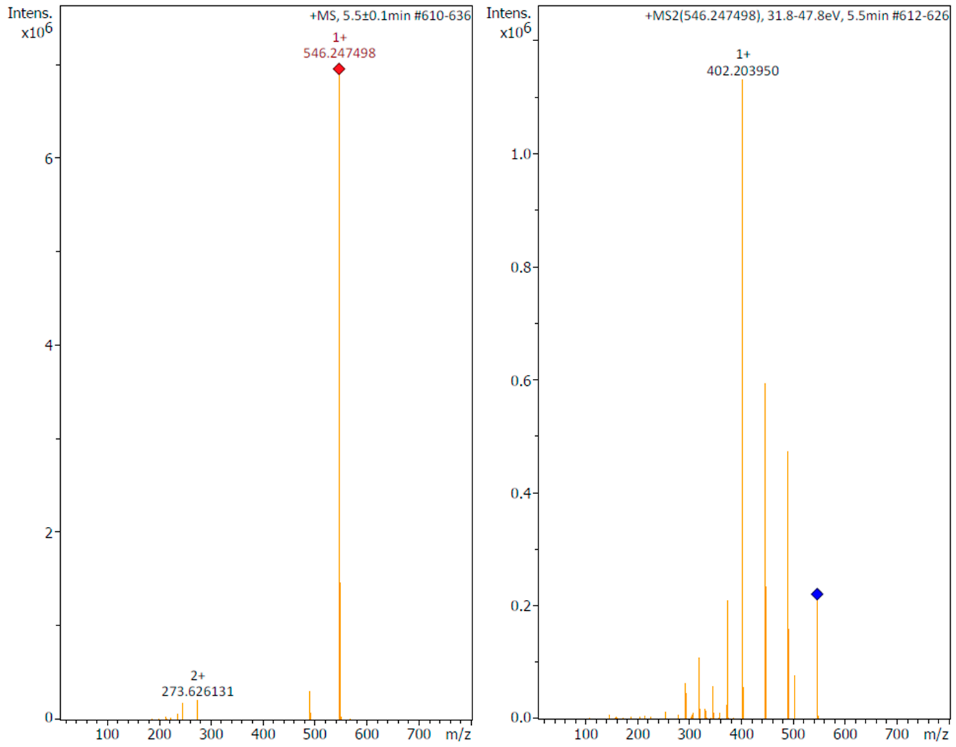Intens.<br>x10^6<br>+MS, 5.5±0.1min #610-636<br>1+<br>546.247498<br>2+<br>273.626131<br>m/z<br>Intens.<br>x10^6<br>+MS2(546.247498), 31.8-47.8eV, 5.5min #612-626<br>1+<br>402.203950<br>m/z

**Risdiplam**		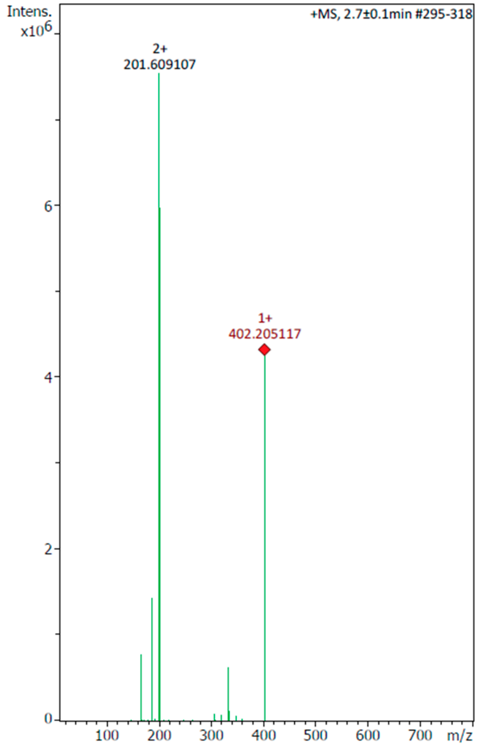 Intens. x10^6 +MS, 2.7±0.1min #295-318 2+ 201.609107 1+ 402.205117 m/z 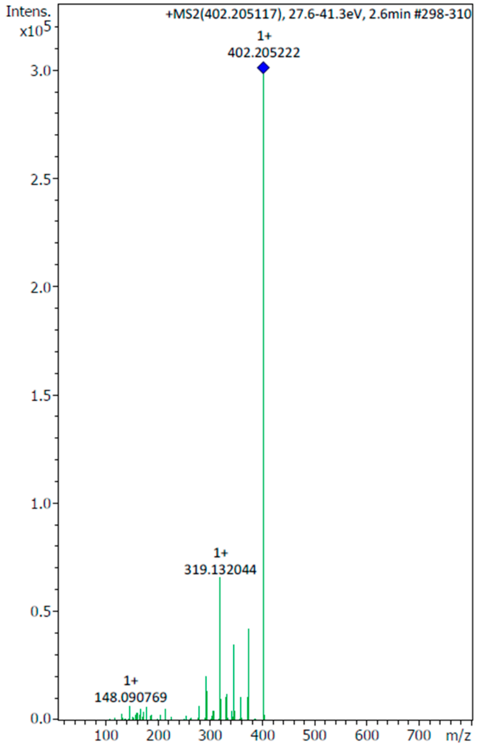 Intens. x10^5 +MS2(402.205117), 27.6-41.3eV, 2.6min #298-310 1+ 402.205222 1+ 319.132044 1+ 148.090769 m/z

**28**			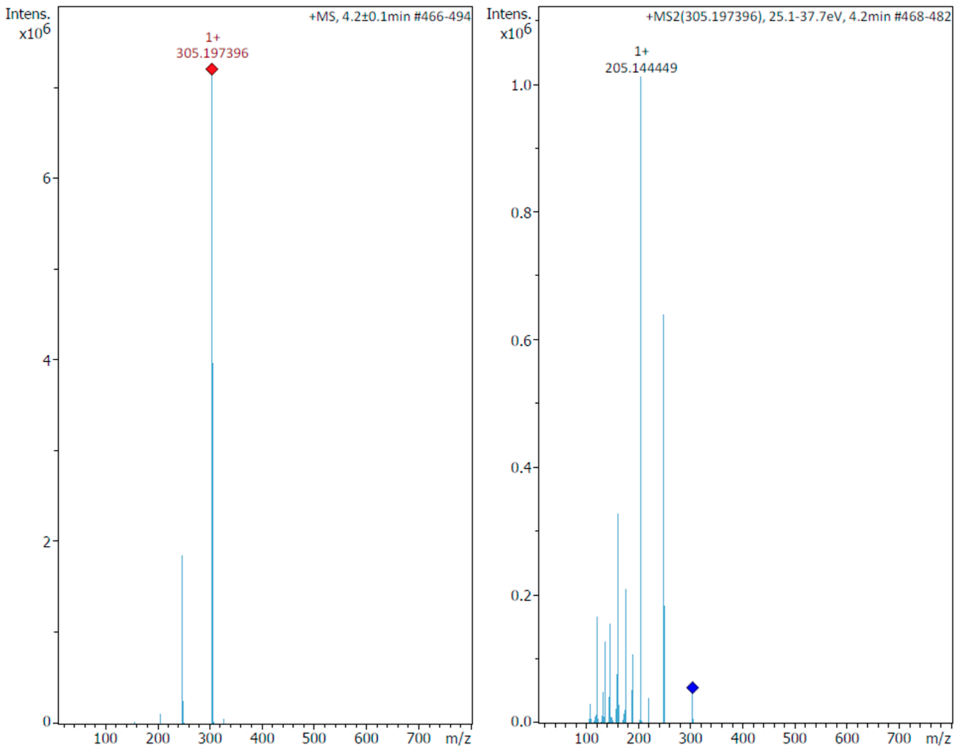

Intens.
x10^6
+MS, 4.2±0.1min #466-494
1+
305.197396
m/z
+MS2(305.197396), 25.1-37.7eV, 4.2min #468-482
1+
205.144449

**30**			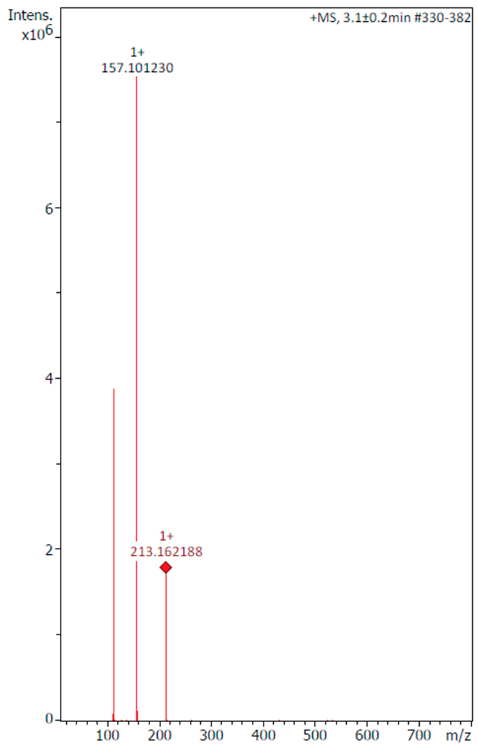 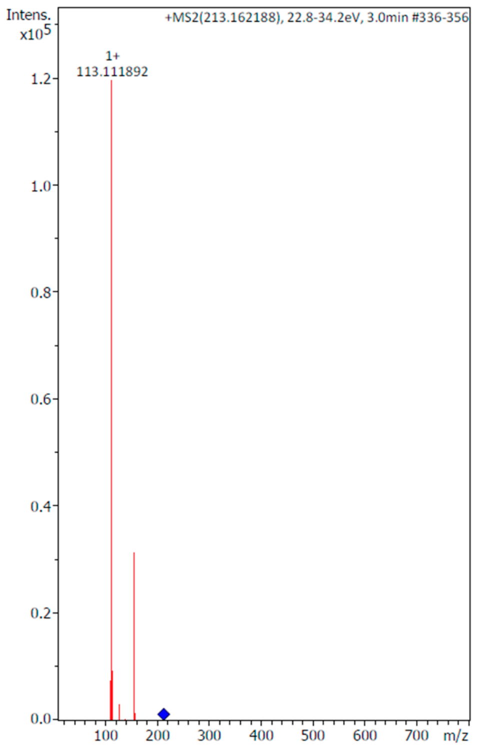

Intens.
x10^6
+MS, 3.1±0.2min #330-382
1+
157.101230
1+
213.162188
m/z

Intens.
x10^5
+MS2(213.162188), 22.8-34.2eV, 3.0min #336-356
1+
113.111892
m/z

**31**			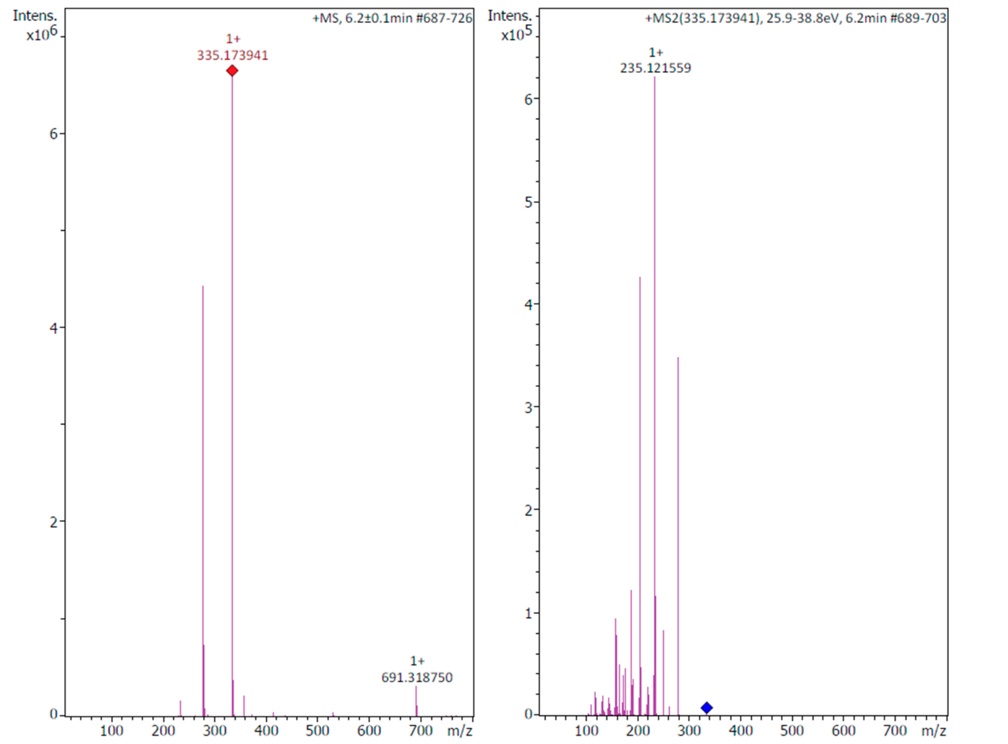

Intens.
x10^6
+MS, 6.2±0.1min #687-726
1+
335.173941
1+
691.318750
m/z
Intens.
x10^5
+MS2(335.173941), 25.9-38.8eV, 6.2min #689-703
1+
235.121559
m/z
